# Genetic and epigenetic variation among inbred mouse littermates: identification of inter-individual differentially methylated regions

**DOI:** 10.1186/s13072-015-0047-z

**Published:** 2015-12-12

**Authors:** Harald Oey, Luke Isbel, Peter Hickey, Basant Ebaid, Emma Whitelaw

**Affiliations:** Department of Genetics, La Trobe Institute for Molecular Science, La Trobe University, Bundoora, Melbourne, VIC 3086 Australia; University of Queensland Diamantina Institute, Translational Research Institute, Princess Alexandra Hospital, Brisbane, QLD 4102 Australia; Bioinformatics Division, The Walter and Eliza Hall Institute of Medical Research, 1G Royal Parade, Parkville, VIC 3052 Australia

**Keywords:** Metastable epiallele, Genetic variation, Inbred mice, DNA methylation

## Abstract

**Background:**

Phenotypic variability among inbred littermates reared in controlled environments remains poorly understood. Metastable epialleles refer to loci that intrinsically behave in this way and a few examples have been described. They display differential methylation in association with differential expression. For example, inbred mice carrying the *agouti viable yellow* (*A*^*vy*^) allele show a range of coat colours associated with different DNA methylation states at the locus. The availability of next-generation sequencing, in particular whole genome sequencing of bisulphite converted DNA, allows us, for the first time, to search for metastable epialleles at base pair resolution.

**Results:**

Using whole genome bisulphite sequencing of DNA from the livers of five mice from the *A*^*vy*^ colony, we searched for sites at which DNA methylation differed among the mice. A small number of loci, 356, were detected and we call these inter-individual Differentially Methylated Regions, iiDMRs, 55 of which overlap with endogenous retroviral elements (ERVs). Whole genome resequencing of two mice from the colony identified very few differences and these did not occur at or near the iiDMRs. Further work suggested that the majority of ERV iiDMRs are metastable epialleles; the level of methylation was maintained in tissue from other germ layers and the level of mRNA from the neighbouring gene inversely correlated with methylation state. Most iiDMRs that were not overlapping ERV insertions occurred at tissue-specific DMRs and it cannot be ruled out that these are driven by changes in the ratio of cell types in the tissues analysed.

**Conclusions:**

Using the most thorough genome-wide profiling technologies for differentially methylated regions, we find very few intrinsically epigenetically variable regions that we term iiDMRs. The most robust of these are at retroviral elements and appear to be metastable epialleles. The non-ERV iiDMRs cannot be described as metastable epialleles at this stage but provide a novel class of variably methylated elements for further study.

**Electronic supplementary material:**

The online version of this article (doi:10.1186/s13072-015-0047-z) contains supplementary material, which is available to authorized users.

## Background

Phenotypic variation in traits like weight and size within inbred mouse colonies has intrigued geneticists for decades [1, 2]. Inbred mice are presumed to be virtually isogenic, and observed variation, therefore, attributable to other factors such as stochastic or environmental events. The precise mechanisms underlying such phenotypic variation are still unclear but some of the variability is likely to be reflected in, and possibly driven by, the epigenome and some is likely to be driven by genetic differences. Human twin studies have shown that epigenomes differ slightly within monozygotic twin pairs [3, 4] but the significance of these differences remains unclear. While monozygotic twins arise from the same zygote, littermates in inbred mouse colonies arise from independent gametes, providing opportunities for genetic differences that result from germline mutations.

Some parts of the genome, such as the telomeres, are known to be variable in length between inbred littermates [5–7]. It has also been shown that some DNA copy number variants persist, despite careful inbreeding [8]. Spontaneous germline mutations will also occur. In humans, whole genome sequencing of trios has been used to estimate that such mutations occur at a rate of 1.20 × 10^−8^ mutations per base per generation [9]. While the corresponding rate in mice was previously believed to be significantly higher [10], recent estimates suggest they are similar [11, 12]. Now, whole genome sequencing can be used to investigate such variation directly. This technology has recently been used to characterize the genomes of some common inbred mouse strains, revealing extensive genetic variation between strains [13]. However, the extent of variation within an inbred strain has not previously been investigated using a whole genome approach.

The *A*^*vy*^ mouse line has been used as a model of epigenetic metastability for many years [14–19]. The founder mouse was discovered 50 years ago in a litter from a C3H/HeJ colony because of its unexpected yellow coat [20]. An intracisternal A particle (IAP) retrotransposon was found to have integrated upstream of the *agouti* gene. The original mouse was backcrossed for many generations to C57BL/6J, and has been maintained on that background in the heterozygous state (*A*^*vy*^/*a*). Littermates range in colour from yellow, through mottled (yellow and brown patches) to pseudoagouti (brown). The coat colour inversely correlates with the DNA methylation state of a promoter within the IAP LTR (long terminal repeat) [18, 21]. The methylation state of the locus within an individual is conserved across tissue types suggesting establishment very early in embryonic development [22]. When active, this promoter drives constitutive transcription of *agouti* and results in a yellow coat. This locus is one of only three or four classic murine metastable epialleles, in which a variable phenotype correlates directly with epigenetic state [22–24]. More recently, it has been proposed that such loci are relatively frequent, in the thousands [25, 26].

We have sequenced the genomes of two littermates from the *A*^*vy*^ mouse colony, one with a yellow coat and one with a pseudoagouti coat, and searched for differences between the two, both at the *A*^*vy*^ locus, and genome-wide, and confirm that genetic differences are unlikely to be involved in the variable coat colour. To discover novel loci that display epigenetic metastability, we used whole genome bisulphite sequencing (WGBS) of the livers of five *A*^*vy*^*/a* mice and searched for regions of significant variability in DNA methylation. We found a small number of loci that behave like metastable epialleles, the most robust are associated with the ERV family of retrotransposons. Most other variable loci are associated with regions identified by others as tissue-specific DMRs, i.e. they display variable DNA methylation across tissues [27].

## Results

### Whole genome sequencing

Whole genome sequencing was carried out using the Illumina sequencing by synthesis technology to 40-fold coverage in two inbred males (one yellow and one pseudoagouti) and the genomes were searched for variants against the C57BL/6J reference genome (mm9). Variants that were identified included both those that differed between the two mice (e.g. heterozygous in one, wild type in the other) (Table 1) and those for which the mice did not differ but differed against the reference genome (i.e. heterozygous or homozygous in both mice) (Table 2). Variant calls at the C3H/HeJ region containing *agouti* were excluded from these counts. No differences between the two mice were seen in this region. Genome-wide, a total of 985 single nucleotide variants (SNVs) were found that differed between the two mice (Table 1; Additional file 1: Table S1) and as expected, the majority of these were located in either intergenic or intronic regions (607 and 324, respectively) (Table 1). Only 11 of the variants were located inside exons, and of these, seven were predicted to result in amino acid changes and four were predicted to be silent (Table 1).

**Table 1:** Variants that are polymorphic between littermates

	Variant count
Intergenic	607
Intronic	324
Exonic	11
Non-synonymous	(7)
Synonymous	(4)
Splice junction	1
Upstream (<2 kb)	18
Downstream (<2 kb)	16
UTR	8
Total	985

Distribution of the variant calls against C57BL/6J reference genome that differs between the two *A* ^*vy*^ littermates. The genetic differences between the Yellow and the Pseudoagouti mouse are divided relative to their genic positions. Exonic mutations have been subdivided into those that are synonymous and those that are not

**Table 2:** Polymorphic variants in the *A* ^*vy*^ colony that are shared by littermates

	Both mice heterozygous	Both mice homozygous
Intergenic	734	2926
Intronic	345	1891
Exonic	21	49
Non-synonymous	(12)	(19)
Synonymous	(7)	(30)
Splice junction	0	9
Upstream (<2 kb)	7	79
Downstream (<2 kb)	11	65
UTR	12	36
Total	1130	5055

Distribution of variant calls against C57BL/6J for which the *A* ^*vy*^ littermates have the same zygosity. The genetic differences between C57BL/6J and the two sequenced mice are divided relative to their genic positions. Exonic mutations have been subdivided into those that are synonymous and those that are not

With respect to the variants that did not differ between the two mice but differed from the reference genome, while such variants are not expected to account for phenotypic variation between the sequenced littermates, the heterozygous variants are likely to be polymorphic within the colony. Most of the homozygous variants are likely to represent mutations that have arisen and spread within the *A*^*vy*^ strain.

To ascertain the false discovery rate of the variant calls, a random set of 105 variants (taken from Additional file 1: Table S1) was picked for Sanger sequencing. 96 of these could be PCR amplified and sequencing was carried out in both littermates. Of these 96, 87 were validated using Sanger sequencing (Additional file 1: Table S2). All variants for which one of the two mice was homozygous were found to represent true positives (out of 24 tested) and 34 variants that were heterozygous in one mouse and wild type in the other (out of 36 tested) were confirmed (Additional file 1: Table S2).

The parents of the two sequenced mice were also tested to determine the proportion that represents de novo mutations. Data were obtained for 32 of the 34 variants that were heterozygous in one mouse and wild type in the other (data not shown). Four variants were unique to one of the offsprings (and absent in the parents) and likely to represent germline mutations. This number can be used to obtain a crude estimate of mutation rate (see “Methods”). A mutation rate of 9.9 × 10^−9^ was obtained, which is similar to that reported using whole genome sequencing for humans [9].

The two genomes were also searched for copy number variations (CNVs) and polymorphic retrotransposon insertions. A single large CNV (Additional file 2: Fig. S1) and 10 retrotransposon insertions were identified that differed between the mice. The latter were either L1 or MTA elements (Additional file 3: Fig. S2). With respect to the former, PCR amplification across the breakpoint showed that it was not linked to the *A*^*vy*^ phenotype (*n* = 12, Additional file 2: Fig. S1b). This CNV has previously been reported in the C57BL/6J strain [28].

### Whole genome methylation

**Fig. 1:**
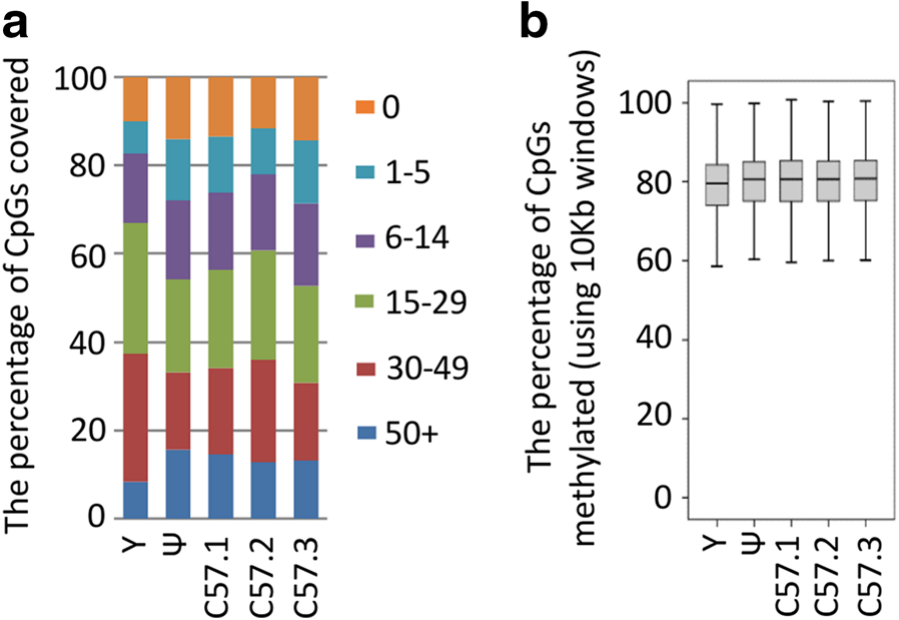
WGBS methylation of the five individuals. **a** The *coloured bars* indicate read depth and the *Y*-axis shows the percent of global CpGs in each category. **b** *Box* and *whisker plot* showing the percentage of all CpGs that are methylated for each of the five individuals, using 10 kb windows across the genome

To identify regions that were differentially methylated among littermates from the *A*^*vy*^ colony, we carried out whole genome bisulphite sequencing on DNA from the livers of five adult males. The *A*^*vy*^ colony was maintained using *A*^*vy*^*/a* crossed to *a/a* mating pairs. Three of the mice were *a/a.* The remaining two were both *A*^*vy*^*/a*, one had a yellow coat (Y) and one a pseudoagouti coat (Ψ). The bisulphite converted genomes were sequenced and more than 70 % of the CpGs were covered by at least 6 reads (Fig. 1a). This is within the recommended range for the identification of differentially methylated regions in WGBS data [29]. The global CpG methylation levels were calculated using 10 kb windows and were found to be similar across the mice, with a median methylation of 80 % (Fig. 1b).

**Fig. 2:**
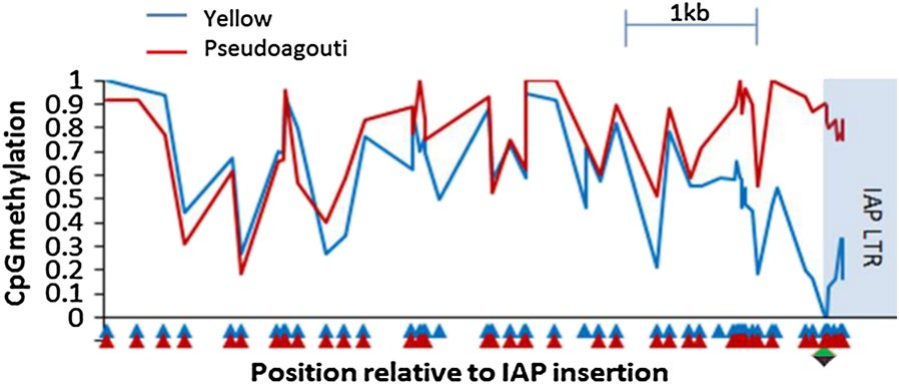
DNA methylation at *A* ^*vy*^. The weighted average DNA methylation levels of single CpG dinucleotides in the yellow mouse (*blue*) and the pseudoagouti mouse (*red*) show changes extending out from the IAP insertion, which is upstream of the *agouti* gene. Data are shown only when more than five reads cover a CpG. Ectopic *agouti* transcripts originate from the LTR element (*green*)

The *A*^*vy*^ locus serves as a positive control for a metastable epiallele with the LTR of the IAP expected to be unmethylated in the Yellow mouse and methylated in the Pseudoagouti mouse. Indeed, this was found to be the case. Interestingly, the difference in methylation was not limited to the IAP long terminal repeat (LTR), but extended approximately 1 kb outside the repeat (Fig. 2), consistent with [30].

### Regions of variable methylation among individuals; inter-individual DMRs, iiDMRs

We then searched the genome for additional regions where the individual mice differed from one another in their methylation. Yellow *A*^*vy*^ mice become obese as adults and the mice used in this study were 22 weeks of age. The Yellow mouse had a bodyweight 1.6 times that of the average of the remaining four (50.7 g versus the average 31.5 g ± 3.1 s.d. for the remainder). To minimize potential confounding effects, the yellow mouse was excluded from the differential methylation calling presented below. For interest, the methylation (and expression) values for the yellow mouse are shown in all figures.

Differentially methylated regions were located by first extracting the CpGs with Chi-squared *P* values that support a difference in methylation (*p* < 0.05). Differentially methylated regions were defined as those loci that had (1) at least six adjacent CpGs (allowing for 10 % of CpGs being uninformative), (2) with a difference of at least 20 % between the weighted averages of the highest and lowest methylated individual and (3), no more than 500 bp between adjacent CpGs. Sites overlapping simple repeats were excluded. A total of 356 regions were identified and clustered by methylation levels (Additional files 1, 4: Table S3, Fig. S3). We call these loci iiDMRs [31], inter-individual DMRs. We noticed that mouse C57.1 was responsible for approximately half of all the identified loci and had consistently higher methylation values at these regions. In the absence of a clear understanding of this, loci that were generated due to high methylation in C57.1 are indicated (Additional file 1: Table S3).

We searched for possible genetic explanations for the differential methylation using the list of 985 and 1130 polymorphisms that were found to be different between the two sequenced mice (Table 1), or were heterozygous in both mice (Table 2), respectively. No iiDMRs directly overlapped with a variant and only two iiDMRs were within 1 kb of a SNV. Similarly, none of the iiDMRs were within 10 kb of the 10 transposable elements that were found to be polymorphic in the colony (Additional file 3: Fig. S2).

**Fig. 3:**
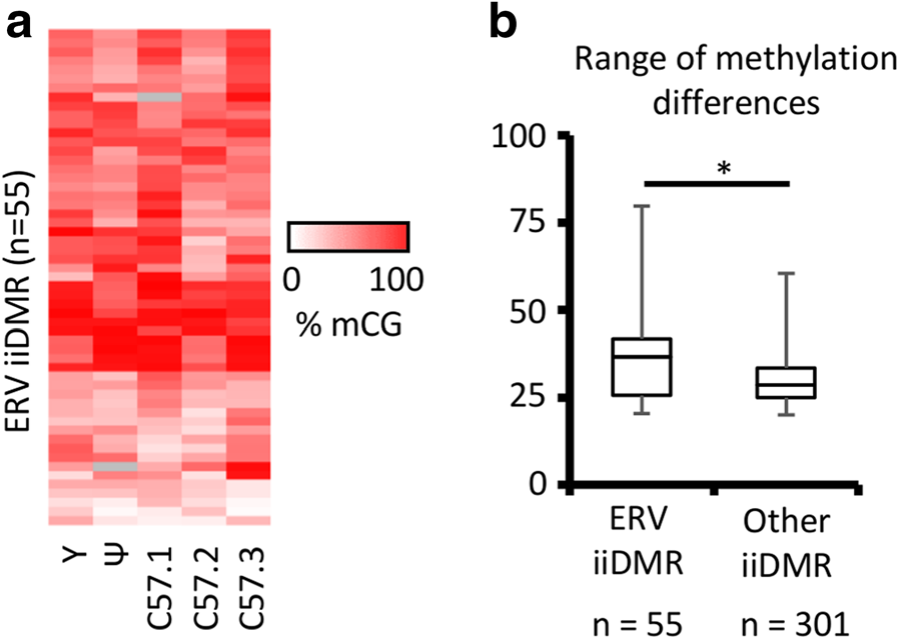
Variable DNA methylation at ERVs. **a** Heatmap representing the 55 iiDMRs overlapping ERV elements. For these sites, the weighted average CpG methylation for each mouse is shown. Unsupervised clustering was performed. Data for the yellow mouse are shown but were not used to identify the differentially methylated sites. **b** The range of methylation at each ERV iiDMR (*n* = 55) for the five mice is shown and compared with that of all 301 iiDMRs generated from Additional file 1: Table S3 after removal of the ERV-associated loci. ERV iiDMRs have a significantly greater range (*T* test, *p* value <0.05)

We initially focused on iiDMRs that overlapped ERVs because the best characterized previously reported metastable epialleles, *agouti viable yellow*, *axin fused* and *Cdk5rap*, are associated with IAPs. We found that 55 of the 365 differentially methylated regions overlapped with ERVs. We refer to these as ERV iiDMRs. The methylation at each region behaved independently with respect to the methylation at other ERV iiDMRs within the same mouse and no single mouse (of all five mice) was consistently more or less methylated at these elements than any other mouse (Fig. 3a; Additional file 1: Table S4). IAPs make up the majority of ERV iiDMRs and RLTR4s may also be overrepresented in this list. RLTR4s are also referred to in the literature as murine leukaemia virus (MLV) type retrotransposons. In general, iiDMRs that overlap with ERVs had a greater range of methylation levels across individuals than the non-ERV iiDMRs (Fig. 3b). Those IAP elements that had an internal sequence had a greater range than lone IAP LTRs (Additional file 5: Fig. S4). The presence of an internal sequence would be expected in recently integrated elements.

**Fig. 4:**
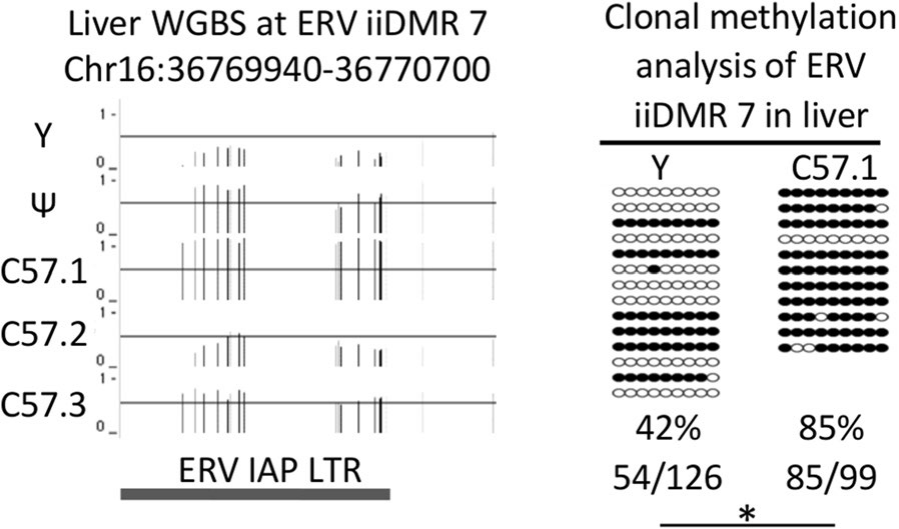
Methylation variability at ERV iiDMR 7 validated using an independent method. A screenshot of the WGBS methylation at ERV iiDMR 7 is shown for the five mice (*left*). On the *Y*-axis 0 represents no methylation, 1 represents 100 % methylation and the *solid lines* indicate the 50 % methylation position. The coordinates of the ERV iiDMR 7 overlaps an IAP LTR, indicated in *dark grey*. Methylation levels from clonal bisulphite sequencing (primer sequences are in Additional file 1: Table S5) on the two extreme samples (yellow and C57.1 mouse DNA) confirmed the differential methylation (*right*). Each sample is represented by at least 11 clones, filled in *circles* represent methylated CpGs from each sequenced clone. *Asterisk* indicates *T* test, *p* value <0.05

Clonal bisulphite sequencing was used to validate methylation levels at one ERV iiDMR designated ERV iiDMR 7, using the same DNA samples used for WGBS and from the two mice with the most extreme methylation states (Fig. 4). This ERV has been reported by others to influence transcription of the *Slc15a2* gene [32].

### Evidence suggesting that these ERV iiDMRs are metastable epialleles

**Fig. 5:**
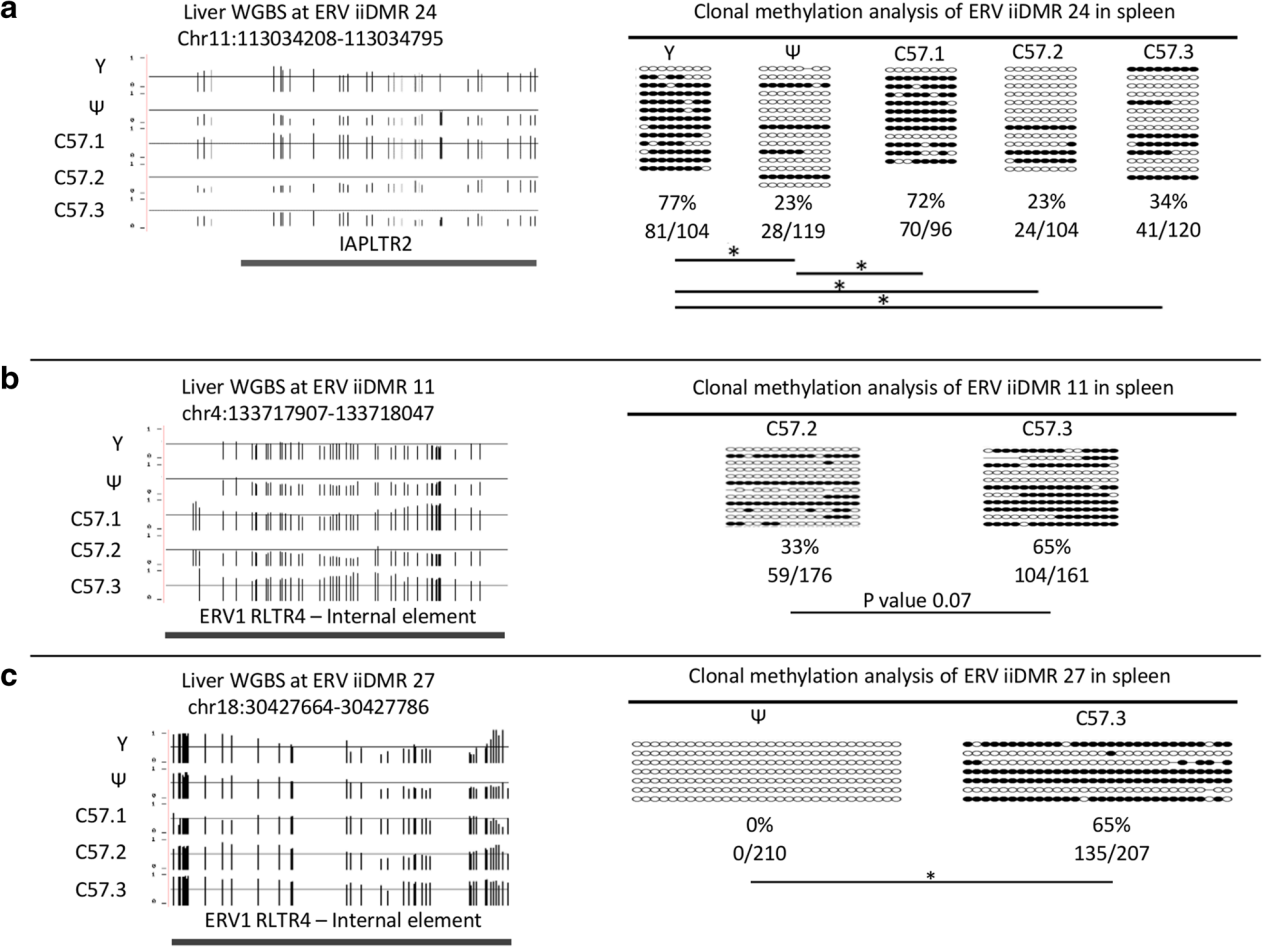
Methylation state at ERV iiDMRs in liver is conserved in spleen. Shown is a UCSC genome browser screen shot of three variably methylated loci, iiDMR 24 (**a**), iiDMR 11 (**b**) and iiDMR 27 (**c**), on the *Y*-axis 0 represents no methylation, 1 represents 100 % methylation and the solid lines indicate the 50 % methylation position. Shown also are the underlying repeat elements. Clonal bisulphite sequencing (primer sequences are in Additional file 1: Table S5) from spleen revealed the same pattern of differential methylation across the five mice for ERV iiDMR 24 and for the two most extremes of methylation for ERV iiDMR 11 and ERV iiDMR 27. Each sample is represented by at least seven clones, filled in *circles* represent methylated CpGs. *Asterisk* indicates *T* test, *p* value <0.05

It has been shown that methylation levels at metastable epialleles correlate across different germ layers within an individual and are, therefore, likely to be set prior to differentiation of the three germ layers [18]. We used clonal bisulphite sequencing to examine the methylation levels for three of the ERV iiDMRs; 24, 11 and 27, in spleen, derived from mesoderm, from the same mice used to generate the liver (endodermal) data. DNA methylation levels across individuals correlated with that found in liver (Fig. 5a–c).

**Fig. 6:**
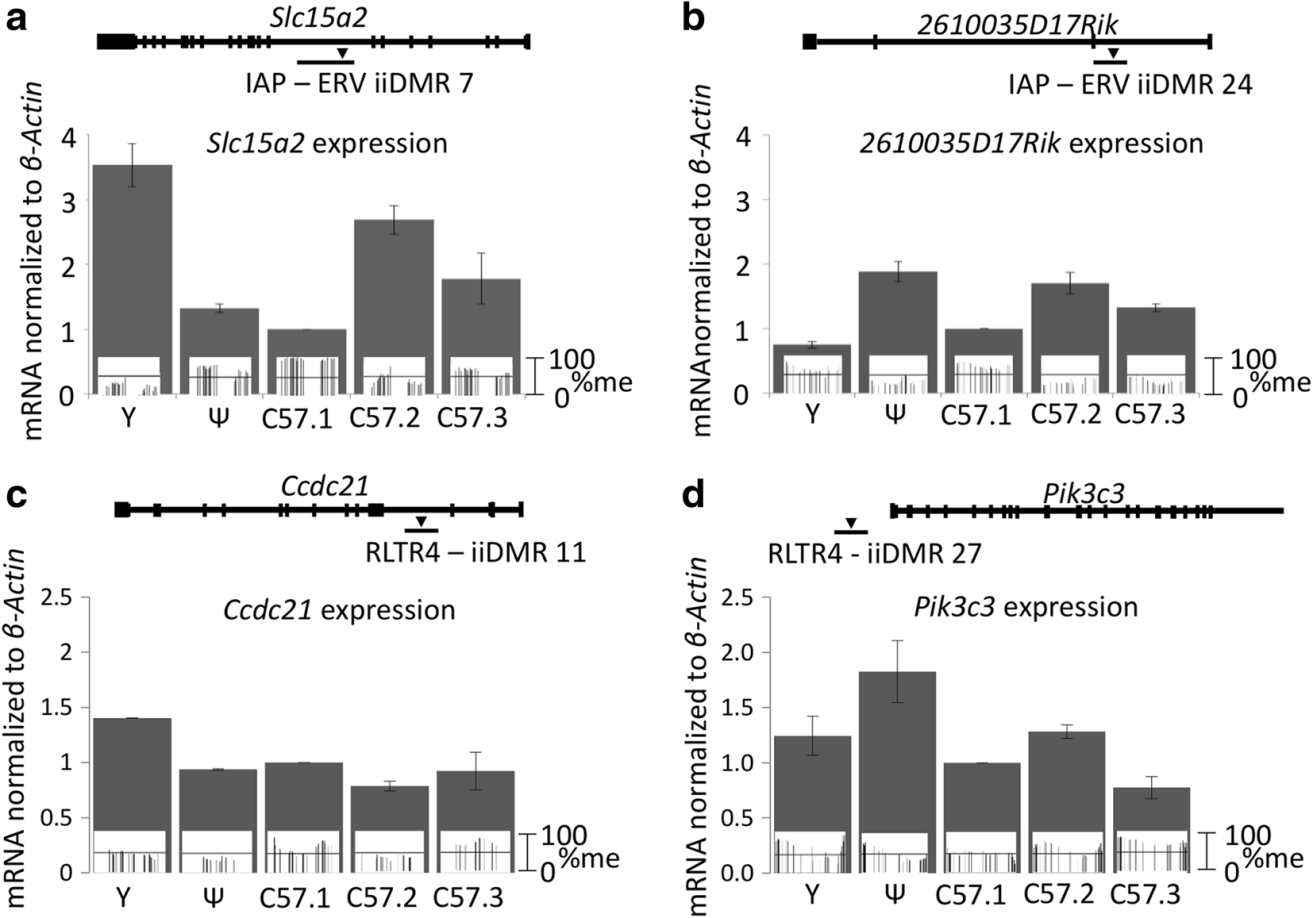
Expression of loci adjacent to ERV iiDMRs. The average expression from four technical replicates is shown for two genes, *Slc15a2* (**a**) and *2610035D17Rik* (**b**), in which ERV iiDMR 7 and ERV iiDMR 24, respectively, are located. The location of each IAP is indicated relative to the exonic and intronic sequences of genes, indicated by *bars* connected by *lines*. Also shown embedded in each expression *bar* is the liver methylation level of the iiDMR taken from Figs. 4 and 5. The average expression from two technical replicates is shown for two genes, *Ccdc21* (**c**) and *Pik3c3* (**d**), associated with ERV iiDMR 11 and ERV iiDMR 27, respectively. The ERV iiDMR 11 RLTR4 is located in intron 3 of *Ccdc21* while the ERV iiDMR 27 RLTR4 is located approximately 5 kb upstream of the *Pik3c3* transcription start site. *Error bars* indicate the SEM for each technical replicate

Two of the classic IAP metastable epialleles, *A*^*vy*^ and *Axin*^*Fu*^, were originally identified because of altered expression patterns among inbred littermates. Using reverse transcriptase quantitative PCR (RTqPCR), we determined the expression of the genes adjacent to the IAP-associated loci, ERV iiDMR 7 and ERV iiDMR 24. The genes are *slc15a2* and *2610035D17Rik*, respectively. As the *Slc15a2* gene is not expressed in liver, we carried out these experiments in spleen. An inverse correlation was seen between the level of methylation and expression of these genes (Fig. 6a, b). This experiment was also carried out on genes adjacent to two ERV iiDMRs associated with RLTR4 elements, ERV iiDMR 11 and ERV iiDMR 27. The methylation state at ERV iiDMR 11 did not inversely correlate with expression of the gene in which it is located, *Ccdc21* (Fig. 6c). An inverse correlation was observed between methylation at ERV iiDMR 27 and expression of the adjacent *Pik3c3* gene (Fig. 6d). These results support the ability of the methodology to identify metastable epialleles and show that for ERV iiDMRs the DNA methylation level often inversely correlates with transcription of an adjacent gene.

### Differentially methylated regions that are not ERVs

Excluding the ERV iiDMRs, 301 other regions satisfied the criteria for a locus that is variably methylated across individuals (Additional file 1: Table S3). Interestingly, many (156/301) of these non-ERV iiDMRs overlapped with short regions that are differentially methylated across tissues within an individual mouse, termed tissue-specific differentially methylated regions, tsDMRs [27]. tsDMRs are conserved regions involved in transcriptional regulation, mainly enhancers.

To validate methylation changes at these non-ERV iiDMRs nine loci were randomly selected and pyrosequencing was used to asses DNA methylation in liver (endodermal), cerebellum (ectodermal) and spleen (mesodermal), representing the three germ layers, from the same five mice. The differential methylation validated in liver at only five out of the nine loci (Additional file 5: Fig. S4a–e). No differences were seen at four of the nine loci in any tissue across the five mice (Additional file 5: Fig. S4f–i). This relatively poor validation rate might be associated with the lack of replicates, even though sequencing was carried out at high coverage [29]. In this study, the experimental design necessitates no biological replicates. Alternatively, around half of these sites might be false positives.

At the five loci that did validate, the differential methylation was not seen in the cerebellum (ectoderm) or the spleen (mesoderm) (Additional file 5: Fig. S4a–e), suggesting that the establishment of these different methylation states does not occur early in development and raising the possibility that cell type ratio changes have occurred in the liver. These loci mostly show a modest range in DNA methylation across individuals compared to the ERV iiDMR group (Fig. 3b). Validation of this group awaits the development of better technologies for detecting small changes in DNA methylation.

## Discussion

This is the first report of the use of whole genome resequencing (approx 40× coverage) to investigate sequence differences between two inbred mouse littermates. Four of the randomly selected variants were absent in both parents, representing likely germline mutations. This is consistent with a mutation rate of 9.9 × 10^−9^, which agrees with previous estimates. In addition, ~2000 SNPs were identified that are either heterozygous in both mice or differ in zygosity between mice, a reminder that in inbred colonies those mutations that have arisen in the recent past are in many cases not fixed in the population despite inbreeding. The relatively small number of SNVs in coding sequences (*n* = 32) and the failure to detect any mutations close to the *A*^*vy*^ allele reassure us that the variable coat colour among *A*^*vy*^ littermates is an epigenetic event.

We found 356 regions that vary in methylation across the five inbred individuals and call these iiDMRs. 55 of these 356 loci overlapped with ERVs and these showed the largest variability in DNA methylation across the mice. Given that the few classic metastable epialleles identified in the mouse prior to this report are linked to transcriptionally active retrotransposons, this is not surprising. Despite the identification of 55 ERV iiDMRs, our statistical calling procedures could not be implemented at all loci, e.g. approximately half of the ~12,000 annotated IAP elements in the mm9 mouse reference genome failed to meet coverage requirements. So 55 ERV iiDMRs are likely to be a twofold underestimate of the ERV iiDMRs in the mouse genome.

We established a statistical method of calling iiDMRs that required six CpGs less than 500 bp apart in an attempt to reduce false positives and this condition will bias our dataset to regions with at least that density of CpGs. The use of biological replicates is recommended for single CpGs resolution [29]. However, we could not use biological replicates (every mouse will, by definition, be different at these loci).

Three of the IAP elements found in this study to be iiDMRs have been reported previously to influence transcription of adjacent gene expression, *Slc15a2* and *Polr1a* [32], and *Cdk5rap1* [23]. Only the last of these has previously been reported to be a metastable epiallele. Most of the ERV iiDMRs reported here are poorly annotated with respect to their ability to influence transcription of adjacent genes but are located well within an interval potentially able to drive gene expression, as exemplified by the IAP at *A*^*vy*^, which lies approximately 100 kb from the *agouti* coding sequence [18].

Clonal bisulfite sequencing using unique primers that flank the repeat element allowed us to reanalyse these ERV iiDMRs in another tissue. This is difficult to accomplish with other targeted approaches that are limited by amplicon size, such as pyrosequencing or methylation-sensitive high-resolution melt analysis, hence the use of clonal bisulfite sequencing. The majority of ERV iiDMRs that were tested here for metastability (i.e. showed the same methylation state in a different tissue and affected expression) turned out to be metastable epialleles.

Others have carried out a bioinformatic screen of IAPs that possess active promoter histone marks, H3K4me3, and identified 143 potential metastable epialleles in the mouse, only three of which overlap with our ERV iiDMR dataset [25]. A follow-up study, by the same group, searched for DNA methylation variability among inbred mice using an IAP enrichment method and report thousands of differentially methylated loci [26], none of which overlap with those reported here. This lack of overlap is likely to be the result of the differences between techniques.

It has previously been reported that the methylation state of two metastable epialleles, *A*^*vy*^ and *Axin*^*Fu*^, are set independently of each other, even when both alleles are present in the same individual [33]. Despite the underlying IAP LTR sequences at these two loci being identical, the programming of each appears to be independent. This is consistent with the ERV iiDMRs identified in this study for which no obvious bias towards hyper or hypo-methylation in any one individual can be detected.

In humans, genetic differences confound the approach used in this study. Despite this, a recent attempt has been made to use genome-wide bisulphite sequencing to identify metastable epialleles. They identified 109 loci that were candidate metastable epialleles with discordant inter-individual DNA methylation. This group of regions was enriched in retrotransposon-derived elements, including LINEs and HERVs [34].

The fact that metastable epialleles produce offspring with a range of phenotypes despite “isogenicity”, might enable genetically closed colonies, e.g. those geographically isolated, to cope with fluctuating environmental conditions. For example, one could envisage a situation in which “yellower” mice are fitter than pseudoagouti littermates, such as a change in habitat from grasses to desert. These animals would maintain the genetic stock during hot periods. On the other hand, such variability in a constant environment is likely to be detrimental, by reducing the number of successful offspring in any litter.

Either way, the small number of such elements in the genome (~50 in our strain) makes it unlikely that metastable epialleles are major drivers of evolution.

Others have identified differences in patterns of retrotransposons across the genomes of 17 inbred strains and found at least 25,000 that are polymorphic [13, 35]. These inbred strains have been maintained as independent colonies for around 100 years (equivalent to ~400 generations). However, without a better understanding of selective pressures, different rates in different mouse strains, different rates for different classes of retrotransposons and the number of breeding pairs used over this period, estimating insertion rates is not feasible.

The identification of 10 polymorphic transposon insertions between two individuals has helped us to rule out such events as contributing to methylation changes but for the reasons stated above, the data cannot be used to accurately estimate insertion rates in this strain. The 10 repeats that were found to differ between the individuals are most likely polymorphic in the colony. Of the ten, five were heterozygous in one mouse and homozygous in the other and the insertions could, therefore, not have happened in the parents of the probands. The remaining five insertions were heterozygous in one individual and absent (i.e. wild type) in the other. As was shown for the analogous germline SNVs (only 4 SNVs of 32 candidates were found to represent germline mutations), most of these are likely to be polymorphic in the colony and the combined insertion rate (L1, MTA and MT2) is, therefore, considerably lower than a maximum of five per generation.

In addition to the retrotransposon-associated iiDMRs, we find a new class of variably methylated loci linked to transcriptional regulatory elements. In general, these loci had a smaller range in methylation across individuals than ERV iiDMRs and the low validation rate at these loci likely reflect the limitations of identifying differentially methylated regions using a single biological replicate. It is possible that some loci in this group are driven by individual differences in cell composition within each mouse’s liver. This limitation extends to all studies using complex tissue and even cells purified using antibodies to surface marker proteins [36]. Even in purified cells it is difficult rule out DNA methylation variation as a reflection of uncharacterised subpopulations [37].

However, it is unlikely that cell type ratio changes underlie large changes in DNA methylation, as each PCR clone and each deep sequencing read represent a single cell and, therefore, changes in DNA methylation would require an equally large change in cell type ratio. Either way, non-ERV iiDMRs represent a novel class of inter-individual DMRs and it will be of interest to study these further.

## Conclusions

Using the most thorough genome-wide profiling techniques for short regions that show differential epigenetic state, we identify approximately three hundred intrinsically epigenetically variable loci and the most robust of these are likely to be associated with recently integrated retroviral elements.

## Methods

### Whole genome resequencing of *A*^*vy*^ littermates

Animal work was conducted in accordance with the Australian code for the care and use of animals for scientific purposes, and was approved by the Animal Ethics Committee of LaTrobe University (project reference number: AEC 12-74). Two male mice heterozygous for the *A*^*vy*^ allele, a yellow and a pseudoagouti, were selected from the *A*^*vy*^ colony and DNA was extracted from tails for whole genome sequencing. Tail DNA was also extracted from the parents and used for downstream validations. Whole genome libraries were prepared using a DNA insert size of 480 bp and sequenced using 2 × 100 bp paired reads on an Illumina HiSeq 2000 by the BGI (Shenzhen, People’s Republic of China). A total of ~7 × 10^8^ paired reads were sequenced for each genome.

The sequenced reads were aligned to the mouse genome (NCBI37/mm9 assembly) using the program BWA, version 0.6.2 [38], and the commands “bwa aln -I -R 500” and “bwa sampe -a 510 -o 1000000”. The mapped reads were coordinate-sorted and PCR duplicates removed using the utility MarkDuplicates from the Picard package (http://picard.sourceforge.net). The reads were then recalibrated by the GATK version 1.6-13 [39] using the tools RealignerTargetCreator (setting −rbs = 10,000,000), IndelRealigner (using indels from the file 20110602-callable-dinox-indels.vcf by Keane et al. [13] to define known alleles), CountCovariates and finally TableRecalibration.

Single nucleotide variants and short indels were extracted by passing the resulting bam-files through the following pipeline utilizing Samtools, BCFtools and VCFtools [40, 41]: samtools mpileup –EDS –g | bcftools view -p 0.99 –vcgN - | vcf-annotate –fill-type -f StrandBias = 0.0001/EndDistBias = 0.0001/MinDP = 14/MaxDP = 100/MinMQ = 25/Qual = 10/MinAB = 6/VDB = 0/GapWin = 3/BaseQualBias = 0.002/MapQualBias = 0.00001/SnpGap = 5/HWE = 0.0001. Variants in which more than 90 % of reads at the locus supported the variant genotype were classified as homozygous while the remaining with at least a frequency of 30 % were classified as heterozygous.

The resulting variants were filtered for overlap with elements annotated as simple repeats with a periodicity <9 in the UCSC Genome Browser [42, 43], homopolymer runs >8 bp (plus 1 bp either end), dinucleotide repeat runs >14 bp (plus 1 bp either end) and regions with an average mapping quality score <40. Additionally, regions where three or more heterozygous variant calls were made within 10 kb of each other, and where the variants also overlapped elements annotated as segmental duplications or annotated repeats, were excluded.

To calculate the proportion of false-positive variant calls, a random set of 102 variants were selected for validation by PCR amplification followed by Sanger sequencing. The distribution of the variant types selected for validation is listed in Additional file 1: Table S2. For six targets, PCR primers could not be designed, or PCR amplification failed to produce amplicons. These were excluded.

The germline mutation rate was extrapolated from the frequency of experimentally validated germline mutations relative to the total number of potential germline mutations from the genome-wide variant calls. Variants on chromosomes X and Y and variants at unplaced contigs located on chromosomes annotated as “Random” (227 Mbp in total) were excluded. Additionally, a total of 258 Mbp was excluded due to repetitiveness, sequence composition or insufficient read coverage, leaving 2136 Mbp in which heterozygous variants could be called for this purpose. The frequency was adjusted for the experimentally derived false-positive discovery rate of 20 %, and a false-negative rate of 23 % (calculated based on variant calls overlapping known variants at the genomic region around Agouti, which is heterozygous for C3H/HeJ) and adjusted for the false-positive and negative rates reported for these variants [13].

CNVs were called by the program Control-FREEC [44] using the settings coefficientOfVariation = 0.05, forceGCcontentNormalization = 1, sex = XY. The resulting calls intersected with genes annotated in the UCSC Genome Browser’s “UCSC Genes” database [43], and calls that overlapped genes were scrutinized for presence of reads and read pairs supporting breakpoints at the termini of each CNV. To visualize such breakpoints, a dataset was created of discordantly mapped read pairs combined with a dataset of soft-clipped reads (identified from the SAM-file CIGAR string), as such reads and read pairs are typically found adjacent to breakpoints.

A CNV that was found using this method was validated by PCR using primers specific to the junction between the tandemly repeated copies using the primers CNV1_F and CNV_R (Additional file 1: Table S5). The distribution of this CNV within the colony was investigated by targeting the junction by PCR in DNA extracted from 12 mice (6 yellow and 6 pseudoagouti). For each template, a control primer (CNV_C), which together with CNV1_F amplifies the wild-type sequence at the 3′ end of the CNV, was used in parallel to verify presence of the template.

To locate transposon insertions that were polymorphic between the two genomes we used the tool RetroSeq [45] with the options –discover -align -len 50 -q 28 –unmapped and –call -reads 10 -depth 100 -hets -q 28. Three separate instances of the program were run with different transposon consensus sequences obtained from RepBase [46] used as input. For endogenous retroviruses (ERV) we used those sequences annotated as Endogenous Retrovirus belonging to the taxon *Mus musculus*. For long interspersed repeat 1 elements (L1), we used sequences annotated as L1 belonging to the taxon *Mus musculus*, plus the following accessions: L1Md_F_5end, L1Md_Gf_5end, L1_Mus1_3end, L1_Mus2_3end. Finally, insertions were also called against the Mammalian apparent LTR retrotransposon (MaLR)-related MTa repeats using the accessions MT2A, MT2B, MT2B1, MT2B2_LTR, MTA, MTAI, MTA_Mm_LTR, MTB, MTB_Mm_LTR, MTC and MTC_I. During the final calling step, putative insertions that overlapped repeats annotated in the UCSC RepeatMasker track as ERVK, L1 and ERVL or MaLR, respectively, were filtered out.

The zygosity of each predicted insertion was then determined by carefully scrutinizing the reads mapped to each locus by visualizing the whole genome datasets in the UCSC Genome Browser [43]. Insertions that were found to have differing zygosity were validated by carrying out local assembly of the discordant and soft-clipped reads that had been mapped to that locus. Assembly was performed by the program Velvet [47] using a hash_length of 50 and –ins_length of 480, and the identity of the inserted element was determined from the resulting contigs by RepeatMasker (http://www.repeatmasker.org).

### Whole genome bisulphite sequencing of *A*^*vy*^ littermates

Whole genome bisulphite sequencing was carried out by Centro Nacional de Análisis Genomico (CNAG, Barcelona, Spain) and the data were processed and mapped, as described previously [48]. An iiDMR was defined as a region with at least six adjacent CpGs with a Chi-squared *p* value <0.05 (allowing for a single CpG without significant *p* value), at most 500 bp spacing each CpG and with a difference of at least 20 % between the weighted averages of the individuals with highest and lowest DNA methylation. Methylation values for each CpG dinucleotide were merged to obtain a single methylation value for each CpG. The weighted average for a region was obtained by dividing each CpG methylation score by the sum of the read coverages across all CpGs in the region followed by multiplying each CpG by the read coverage at that individual CpG and finally adding together each of the adjusted CpG scores to obtain a final score. Those CpGs with less than a 6 read coverage were discarded and values for the remaining CpGs used to identify iiDMRs using custom R scripts (available on request).

### Clonal bisulphite sequencing

Bisulphite treatment was performed on DNA samples purified using phenol–chloroform-extracted DNA. 500 ng of DNA was bisulphite converted using the Qiagen EpiTect Bisulphite Kit (Qiagen, Doncaster, VIC, Australia) and single loci were amplified using primers designed to only amplify bisulfite-converted DNA strands without CpGs in primer sequence. PCR product was purified using the QIAquick PCR purification kit (Qiagen, Doncaster, VIC, Australia), then cloned using the pGEM-T Easy Vector (Promega, Alexandria, NSW, Australia). Clones were sequenced using The BigDye Terminator v3.1 Cycle Sequencing Kit (Life technologies, Mulgrave, VIC, Australia) as per kit instructions. Primers used for bisulphite sequencing are given in Additional file 1: Table S5. To calculate statistical significance, a Student’s *T* test was used to compare the fractions of methylated CpGs for an individual’s bisulfite PCR clones (i.e. the per-clone methylation values) to those of another individual’s clones.

### Pyrosequencing of bisulphite-treated DNA

DNA was extracted by phenol–chloroform followed by ethanol precipitation. Primer design, bisulphite conversion and pyrosequencing were carried out by the Australian Genome Research Facility. Average methylation scores were collected from at least 4 CpGs per locus.

### Reverse transcriptase quantitative polymerase chain reaction

Total RNA was extracted from snap frozen tissues either using TRIzol reagent (Life technologies, Mulgrave, VIC, Australia) or the AllPrep DNA/RNA/Protein kit (Qiagen, Doncaster, VIC, Australia) according to manufacturer instructions. cDNA synthesis was carried out from total RNA using the QuantiTect Reverse Transcription Kit (Qiagen, Doncaster, VIC, Australia) and RTqPCR was performed with the QuantiTect SYBR Green reagent (Qiagen, Doncaster, VIC, Australia). Samples were run on the CFX384 Touch Real-Time PCR Detection System (Biorad, Gladesville, NSW, Australia), with the following conditions: 95 °C 10 min, 39× cycles with 95 °C 15 s then 60 °C 1 min, with a final step of 95 °C 15 s. Relative cDNA abundance was calculated using the ∆∆CT method normalizing to housekeeper gene expression indicated in the figures. Primers are in Additional file 1: Table S5.

## Availability of supporting data

The data sets supporting the results of this article are available in the Gene Expression Omnibus repository, under the accession number GSE72177 (http://www.ncbi.nlm.nih.gov/geo/query/acc.cgi?token=mxqvqeqorbephop&acc=GSE72177).

## Additional files

10.1186/s13072-015-0047-z Variant calls from whole genome sequencing of two Avy mice, one yellow and one pseudoagouti. The variant calls and associated genomic coordinates are relative to the NCBI37/mm9 genome assembly. **Table S2.** Validation of variant calls. A set of 105 SNP variants were selected at random from those that differed between the two mice or were heterozygous in both mice. Of the 96 that could be PCR amplified and sequenced, those that did or did not validate are shown. **Table S3.** Variably methylated regions designated iiDMRs. A list of 356 regions that display differential methylation between inbred individuals, generated from 6 adjacent CpGs that support differential methylation and a difference of at least 20 % between the highest and lowest value. Values from the Yellow excluded from calling regions. **Table S4.** Variably methylated regions that overlap ERV elements, designated ERV iiDMRs. A list of 55 regions that display differential methylation between inbred individuals and overlap with ERV elements from the UCSC mm9 repeatmasker database. Differentially methylated regions were generated from 6 adjacent CpGs that support differential methylation and a difference of at least 20 % between the highest and lowest value. Values from the Yellow mouse were excluded from calling regions. **Table S5.** Primers used in the study.
10.1186/s13072-015-0047-z (a) Read-coverage at a locus where a large gain of ~ 200 Kb of DNA is polymorphic in the Avy colony. Protein-coding genes are illustrated below the graph. (b) A total of six yellow (Y) and six pseudoagouti (P) mice, as well as the two individuals whose genomes were sequenced, were investigated for presence of the CNV using primers amplifying the junction between the copies. For each, a region not affected by the CNV was amplified to confirm presence of the genomic DNA template. A non-template control (N) was also included.
10.1186/s13072-015-0047-z Transposon insertions that differ between littermates. The genomes of two agouti viable yellow mice, one with yellow and one with pseudoagouti coat colour, were sequenced and searched for retrotransposon insertions, relative to the C57/BL6 reference genome (NCBI37/mm9 assembly). Insertions that differed between the two mice are presented below. In the figures, paired deep sequencing reads are presented in the form of red bars (forward reads) and blue bars (reverse reads) connected by black lines (the un-sequenced part of the insert). Each figure is centred on the transposon insertion site, which is usually defined by truncated (soft clipped) reads and flanked by un-paired or discordantly mapped deep sequencing reads.
10.1186/s13072-015-0047-z Candidate differentially methylated regions between littermates. The mm9 genome were searched for sites that had a methylation values significantly different between the Pseudoagouti, C57.1, C57.2 and C57.3 mice with at least 6 adjacent CpGs and a range of at least 20 %. For each site, the weighted average CpG methylation was calculated and used for clustering (unsupervised).
10.1186/s13072-015-0047-z The range of methylation at ERV iiDMRs that overlap with IAP elements that have an internal sequence (n = 17) or are lone IAP LTR elements (n = 10). IAPs with internal sequence elements have a significantly greater range (T-test, p-value > 0.05).
10.1186/s13072-015-0047-z Validation of DNA methylation at random non-ERV iiDMRs. WGBS weighted averages for DNA methylation values are shown for the nine loci chosen for pyrosequencing (a-i). The average pyrosequencing methylation level, from at least 4 individual CpGs from each iiDMR, is shown for liver (L), cerebellum (C) and spleen (S). DNA from these tissues was made using the five mice originally used for WGBS. Methylation levels validated in liver DNA for five of nine loci (a-e).

## Abbreviations

A^vy^: *agouti viable yellow*
CNV: copy number variations
CpG: cytosine guanine dinucleotide sequence
ERVs: endogenous retroviral elements
iiDMRs: inter-individual differentially methylated regions
IAP: intracisternal A particle
LTR: long terminal repeat
RTqPCR: reverse transcriptase quantitative polymerase chain reaction
SNV: single nucleotide variants
tsDMRs: tissue-specific differentially methylated region
WGBS: whole genome bisulphite sequencing
