# Supplementary figures and images for: Genetic and epigenetic variation among inbred mouse littermates: identification of inter-individual differentially methylated regions
Source: Epigenetics Chromatin. 2015 Dec 12;8:54. doi: 10.1186/s13072-015-0047-z (PMC4676890; doi:10.1186/s13072-015-0047-z)

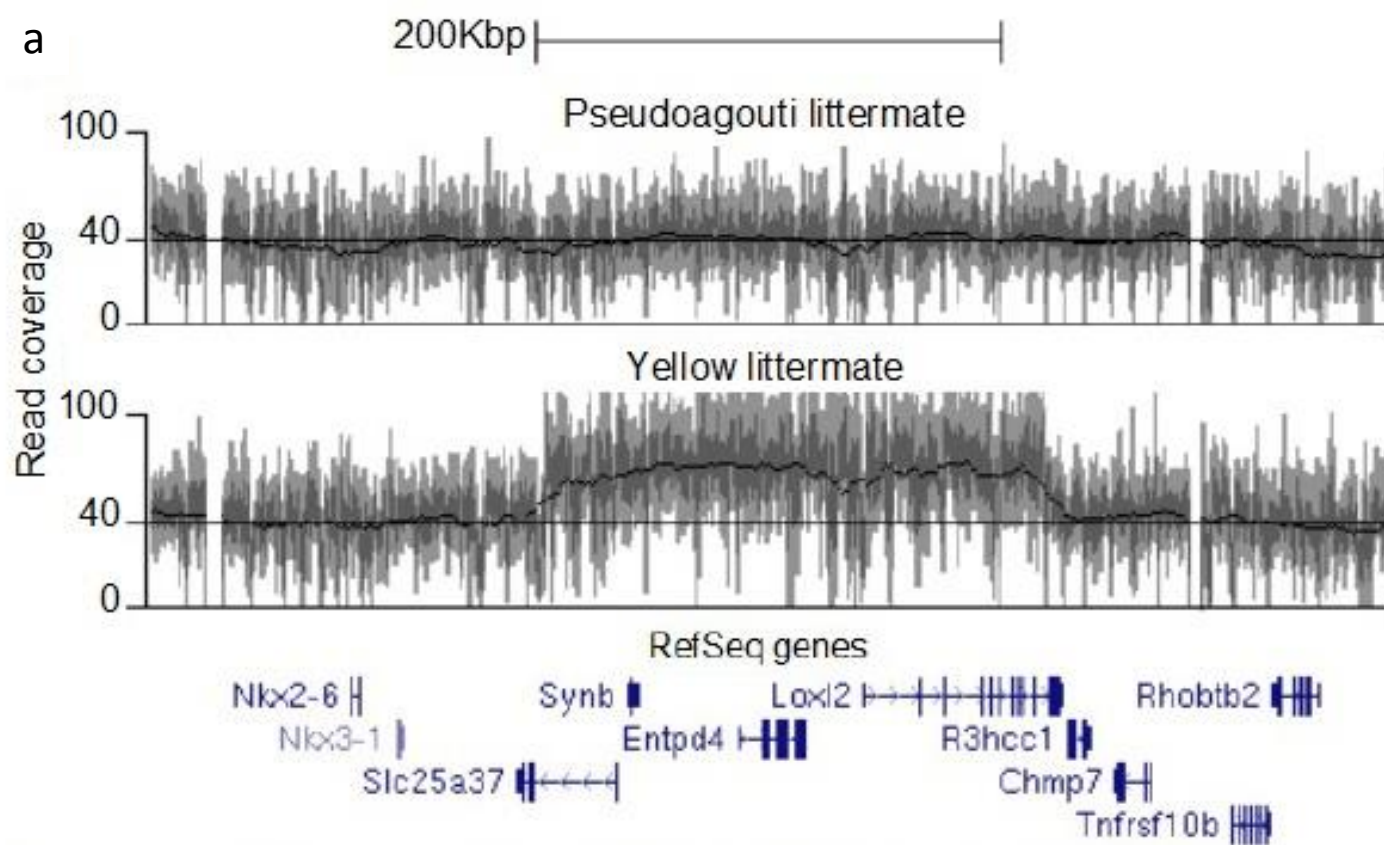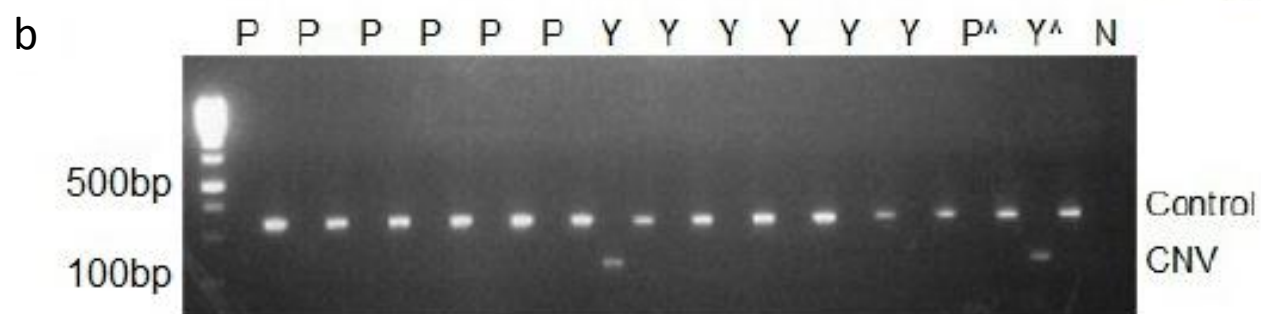

Supplement: Supplementary file 2 — 10.1186/s13072-015-0047-z (a) Read-coverage at a locus where a large gain of ~ 200 Kb of DNA is polymorphic in the Avy colony. Protein-coding genes are illustrated below the graph. (b) A total of six yellow (Y) and six pseudoagouti (P) mice, as well as the two individuals whose genomes were sequenced, were investigated for presence of the CNV using primers amplifying the junction between the copies. For each, a region not affected by the CNV was amplified to confirm presence of the genomic DNA template. A non-template control (N) was also included. [file 13072_2015_47_MOESM2_ESM.pdf]

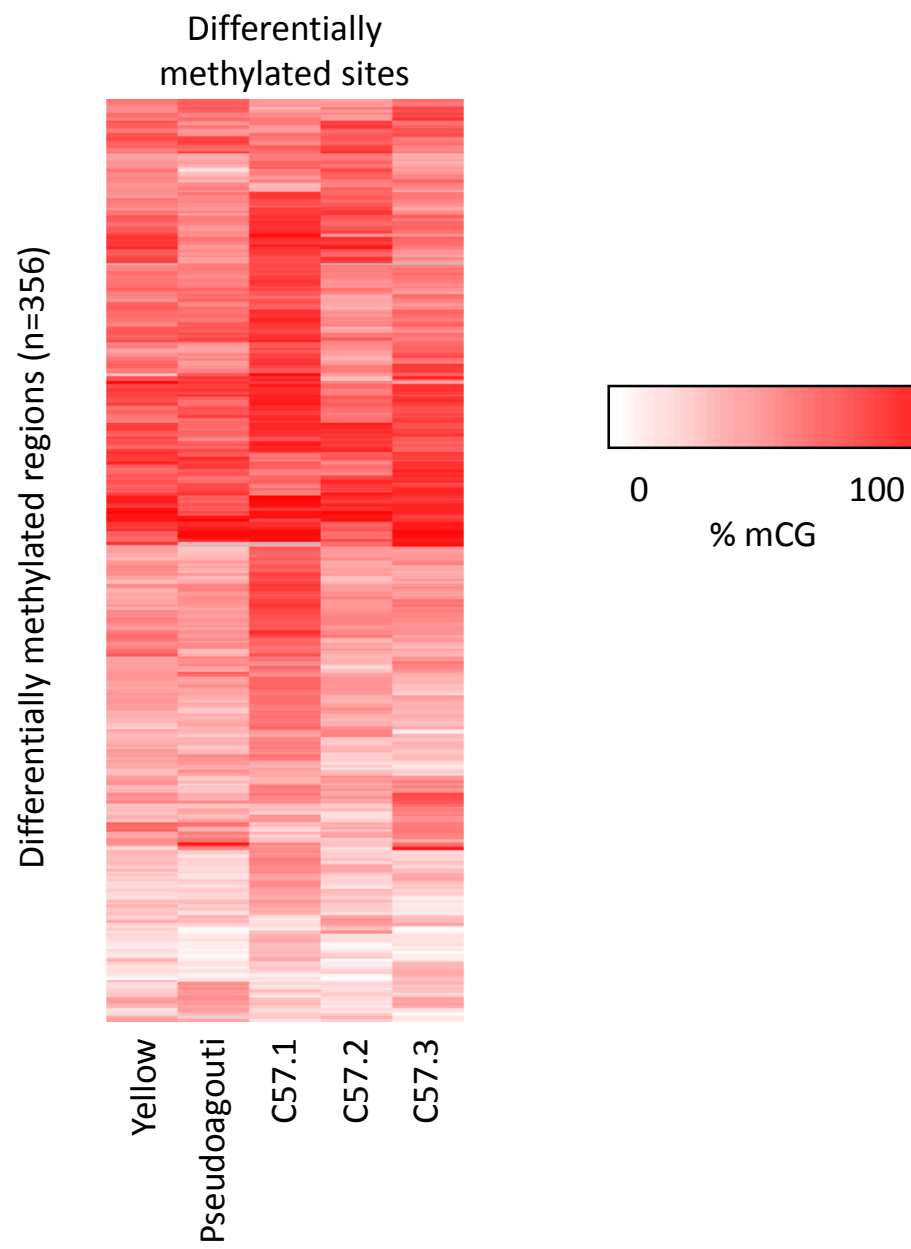

Supplement: Supplementary file 4 — 10.1186/s13072-015-0047-z Candidate differentially methylated regions between littermates. The mm9 genome were searched for sites that had a methylation values significantly different between the Pseudoagouti, C57.1, C57.2 and C57.3 mice with at least 6 adjacent CpGs and a range of at least 20 %. For each site, the weighted average CpG methylation was calculated and used for clustering (unsupervised). [file 13072_2015_47_MOESM4_ESM.pdf]

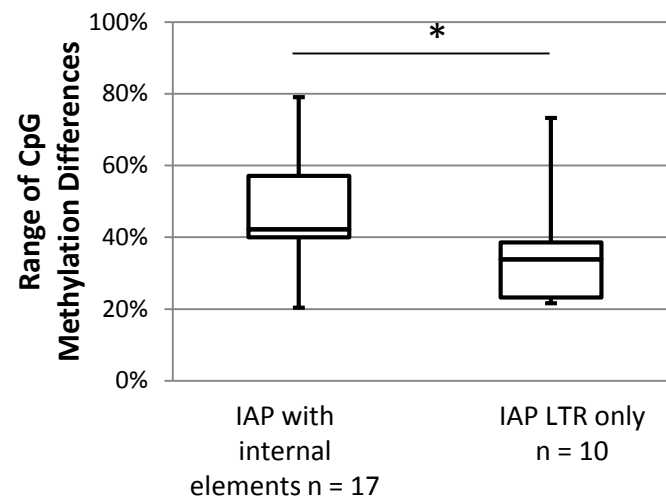

Supplement: Supplementary file 5 — 10.1186/s13072-015-0047-z The range of methylation at ERV iiDMRs that overlap with IAP elements that have an internal sequence (n = 17) or are lone IAP LTR elements (n = 10). IAPs with internal sequence elements have a significantly greater range (T-test, p-value > 0.05). [file 13072_2015_47_MOESM5_ESM.pdf]
