# Supplementary material for: Genetic and epigenetic variation among inbred mouse littermates: identification of inter-individual differentially methylated regions
Source: Epigenetics Chromatin. 2015 Dec 12;8:54. doi: 10.1186/s13072-015-0047-z (PMC4676890; doi:10.1186/s13072-015-0047-z)
Supplement: Supplementary file 3 — 10.1186/s13072-015-0047-z Transposon insertions that differ between littermates. The genomes of two agouti viable yellow mice, one with yellow and one with pseudoagouti coat colour, were sequenced and searched for retrotransposon insertions, relative to the C57/BL6 reference genome (NCBI37/mm9 assembly). Insertions that differed between the two mice are presented below. In the figures, paired deep sequencing reads are presented in the form of red bars (forward reads) and blue bars (reverse reads) connected by black lines (the un-sequenced part of the insert). Each figure is centred on the transposon insertion site, which is usually defined by truncated (soft clipped) reads and flanked by un-paired or discordantly mapped deep sequencing reads. [file 13072_2015_47_MOESM3_ESM.pdf]

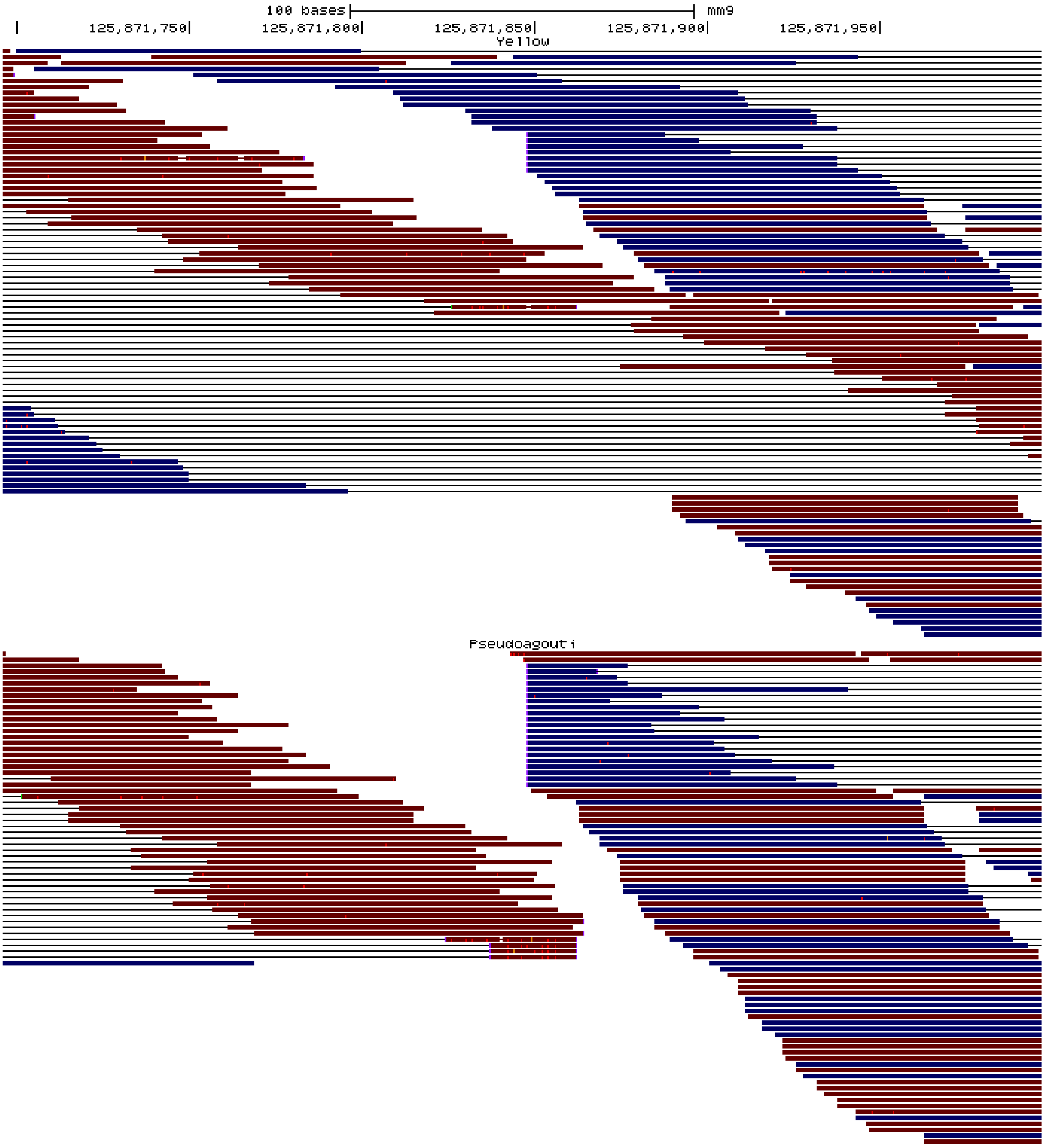

chr1:125,871,697-125,871,997

- L1 insertion
- Yellow mouse heterozygous, pseudoagouti mouse homozygous

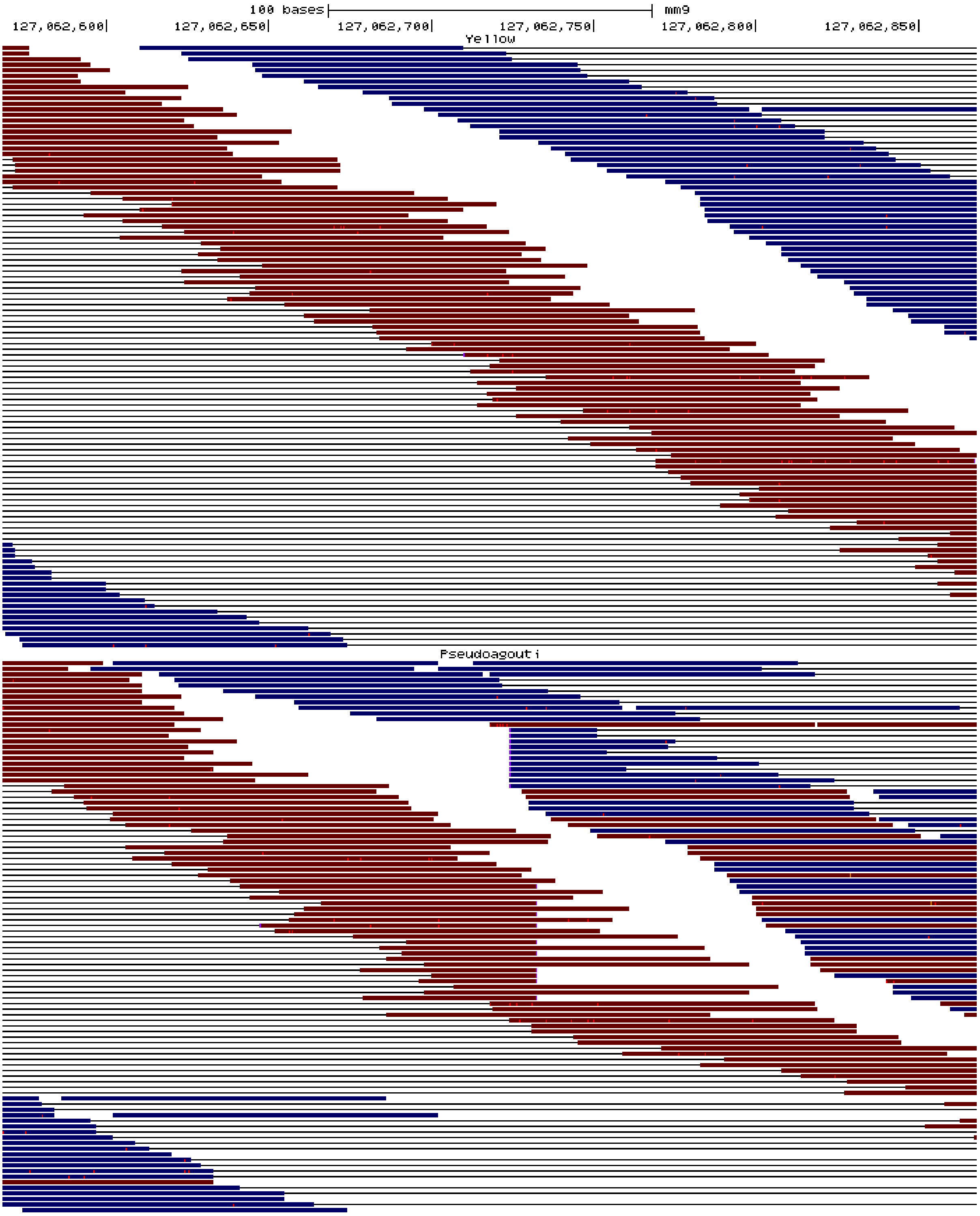

chr1:127,062,569-127,062,868

- MT2 insertion
- Yellow mouse wildtype, pseudoagouti mouse heterozygous

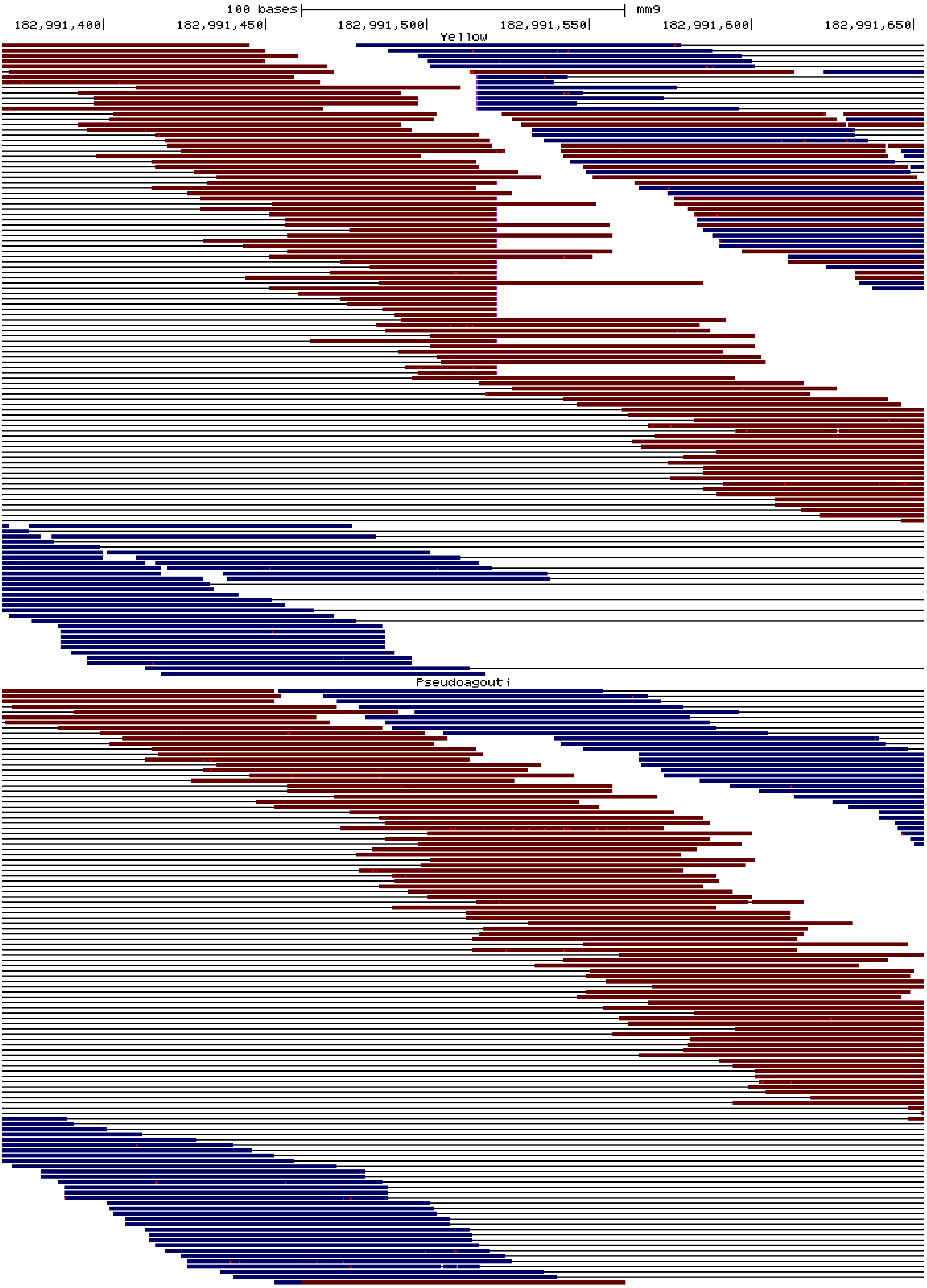

chr1:182,991,423-182,991,654

- MT2 insertion
- Yellow mouse heterozygous, pseudoagouti mouse wildtype

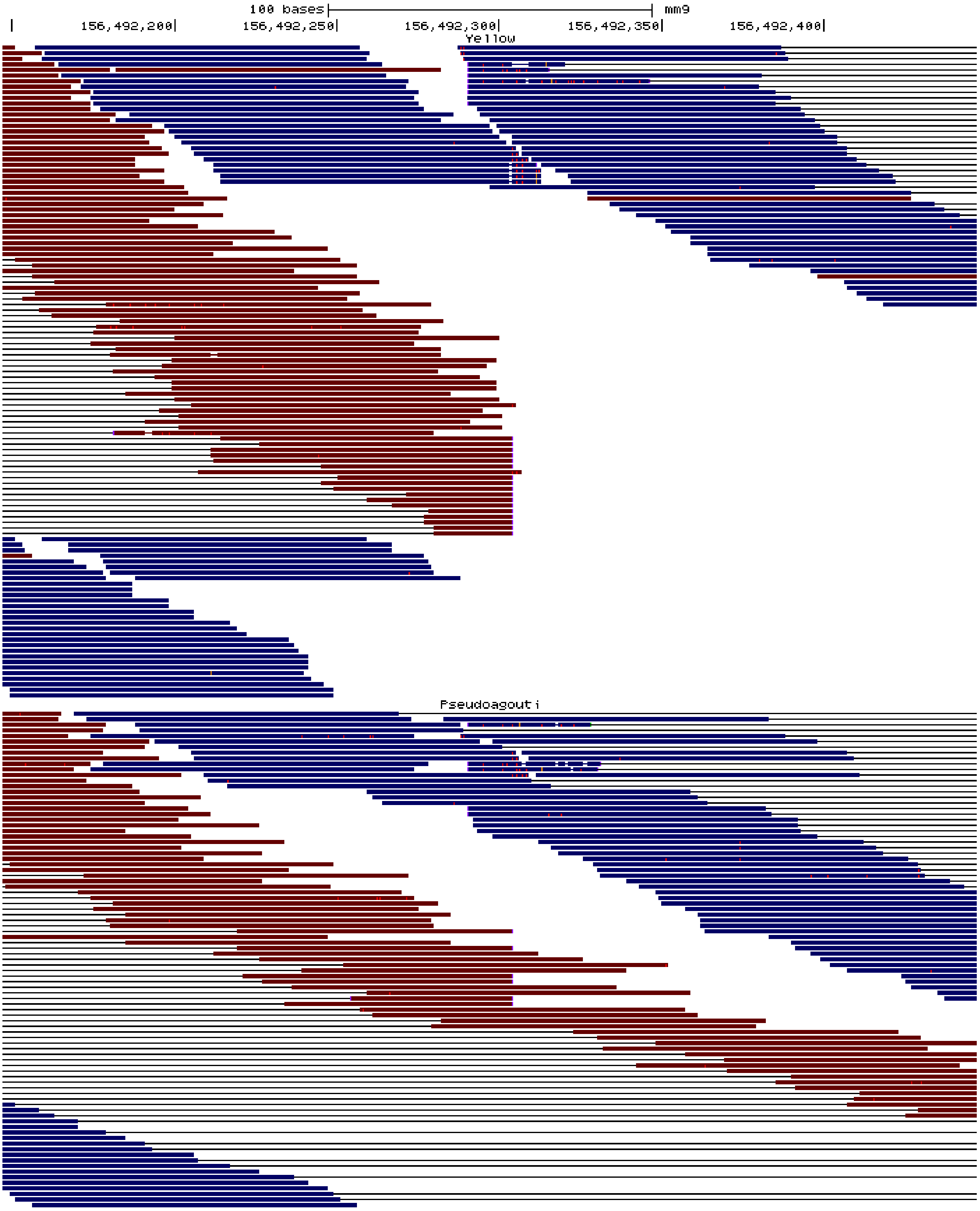

chr3:156,492,148-156,492,447

- L1 insertion
- Yellow mouse homozygous, pseudoagouti mouse heterozygous

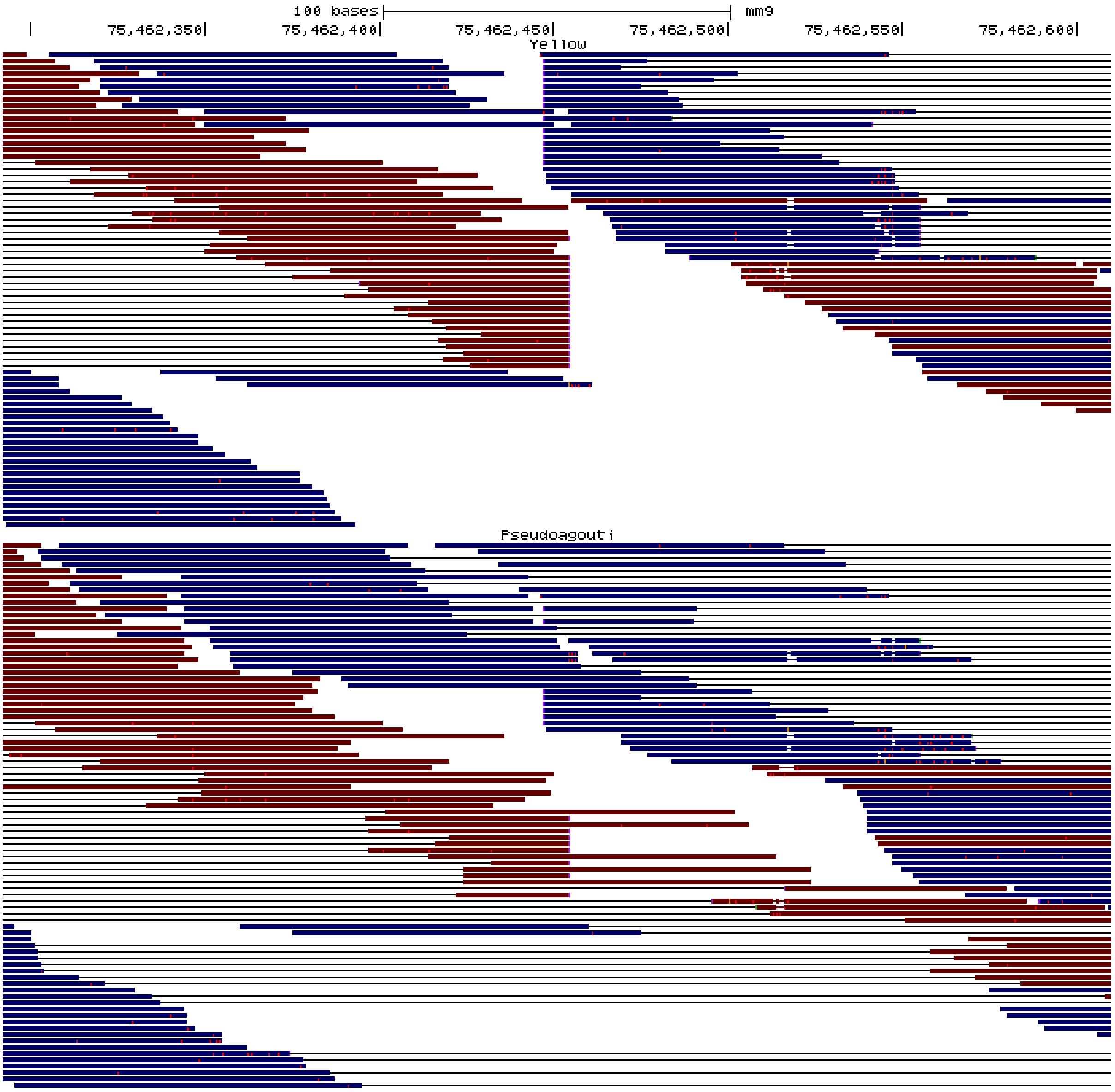

chr5:75,462,293-75,462,610

- MTA insertion
- Yellow mouse homozygous, pseudoagouti mouse heterozygous

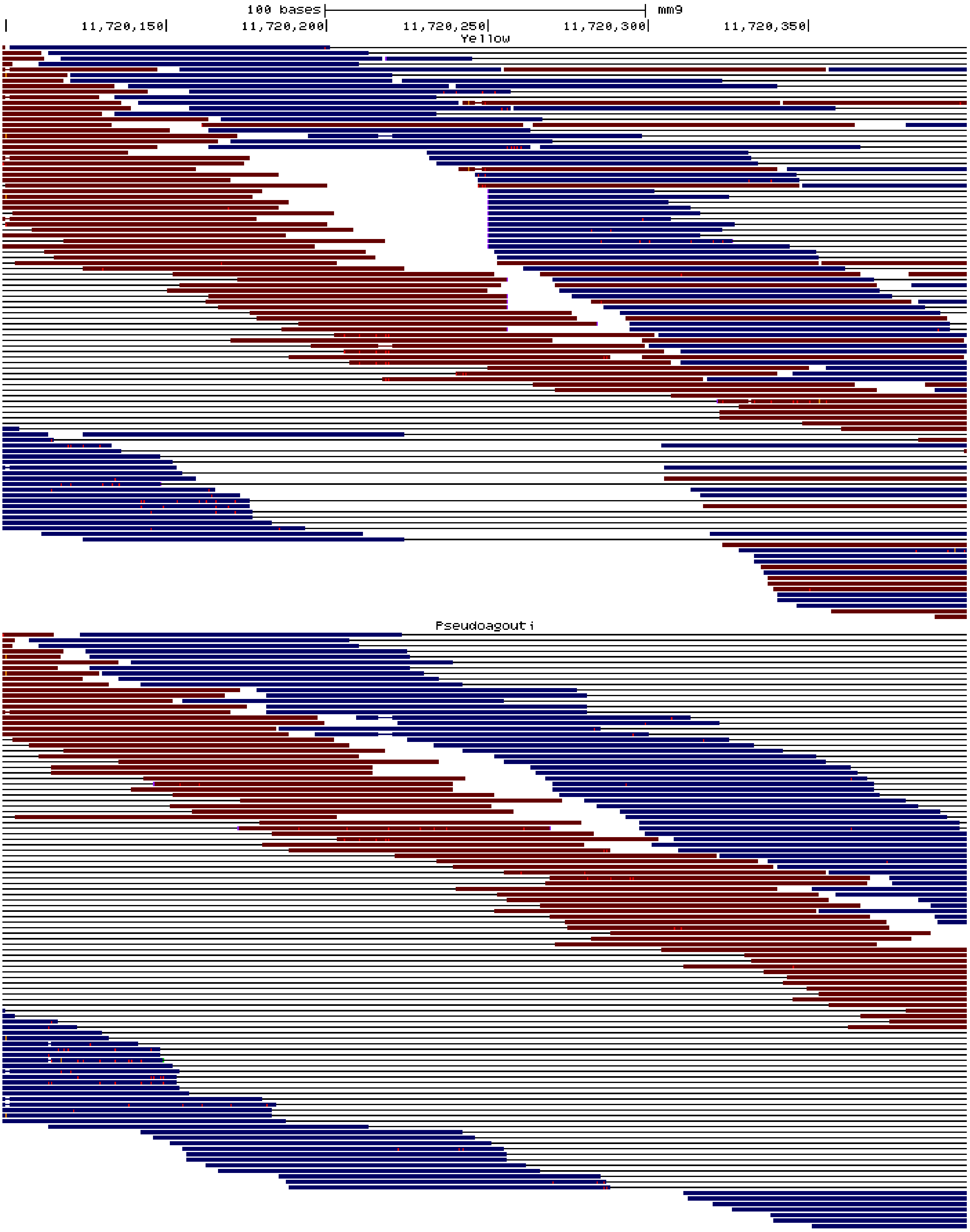

chr6:11,720,100-11,720,399

- MTA insertion
- Yellow mouse heterozygous, pseudoagouti mouse wildtype

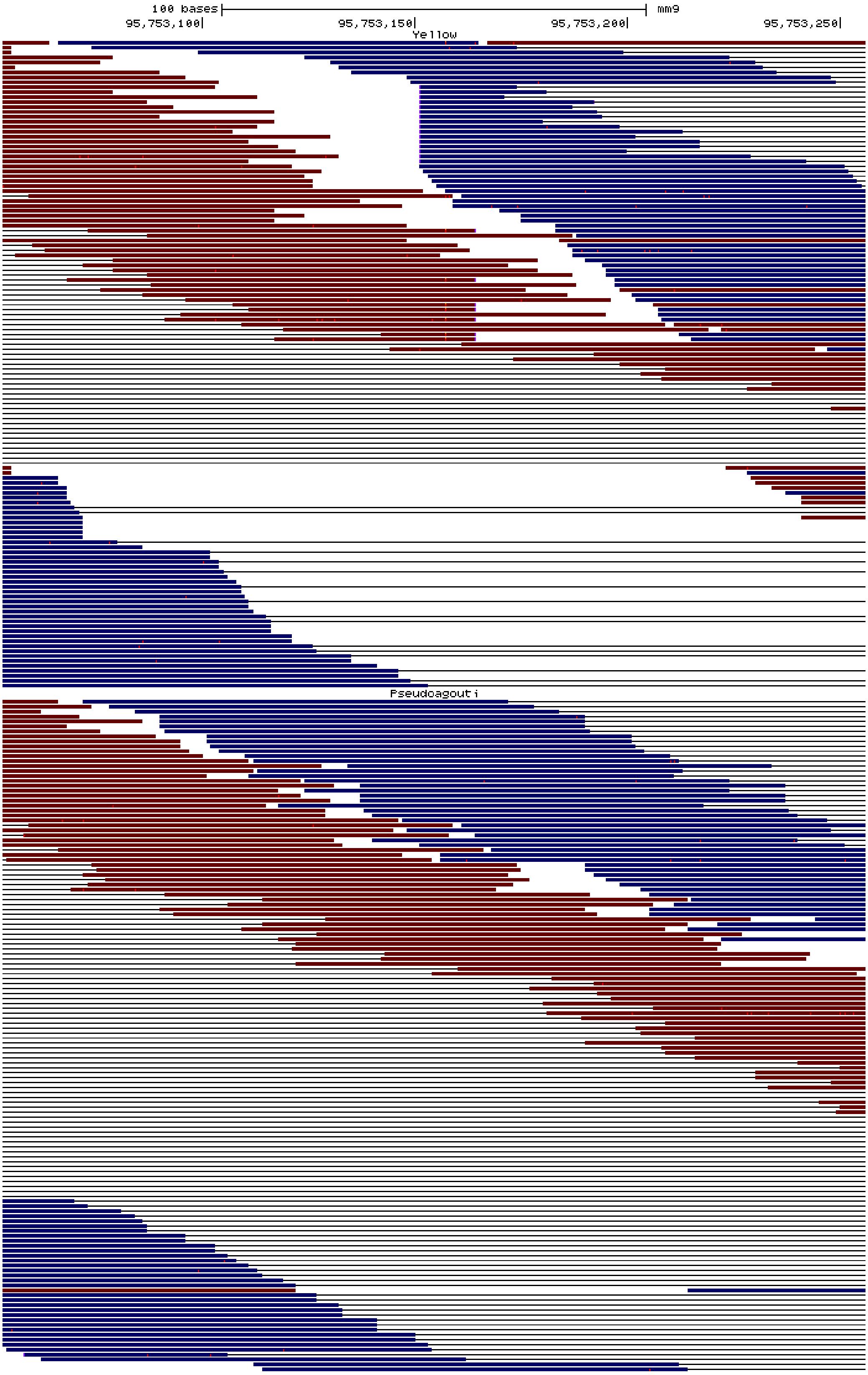

chr9:95,753,054-95,753,256

- MTA insertion
- Yellow mouse heterozygous, pseudoagouti mouse wildtype

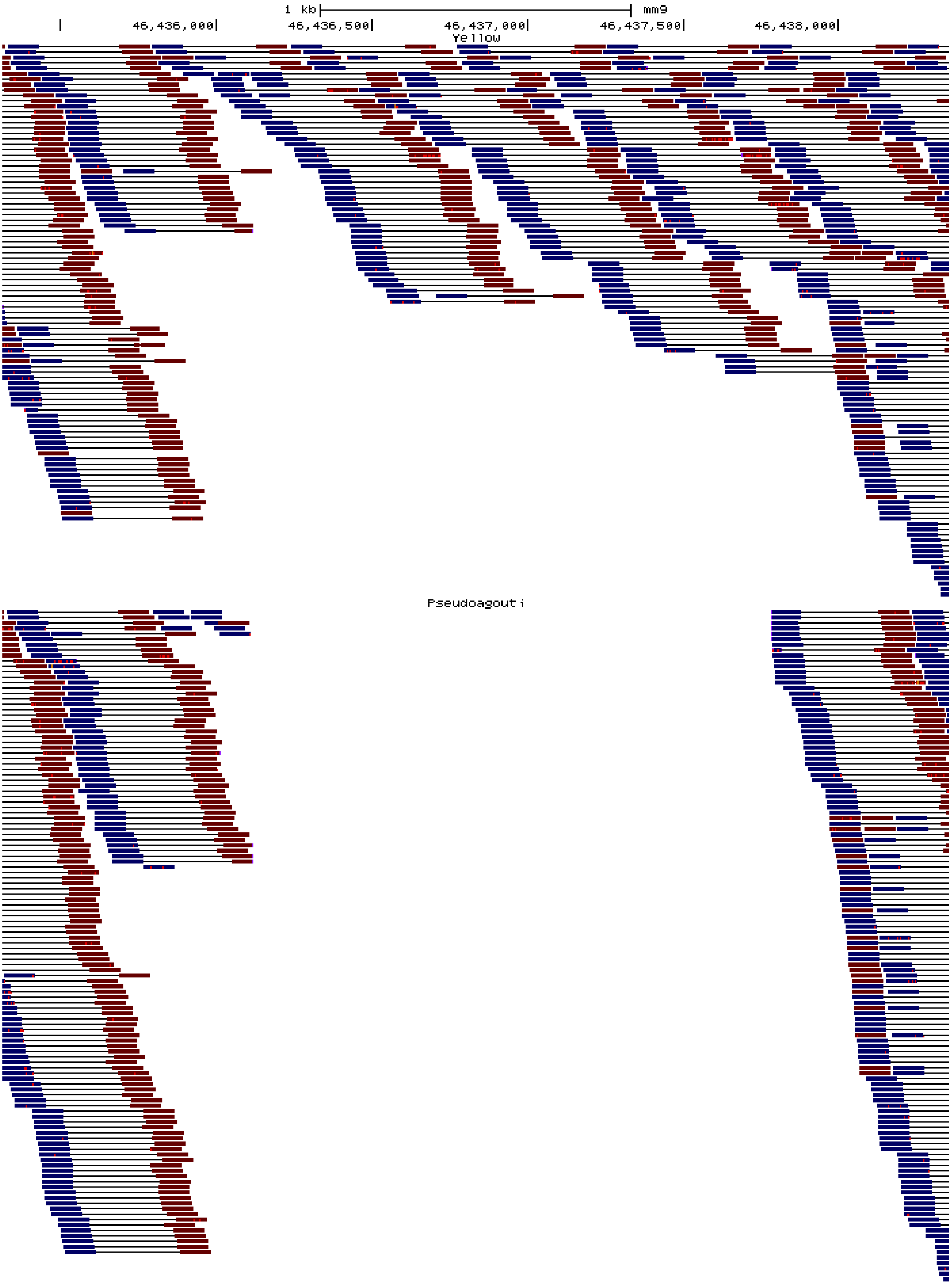

chr10:46,435,316-46,438,353

- L1 insertion combined with a small deletion
- Yellow mouse heterozygous, pseudoagouti mouse homozygous

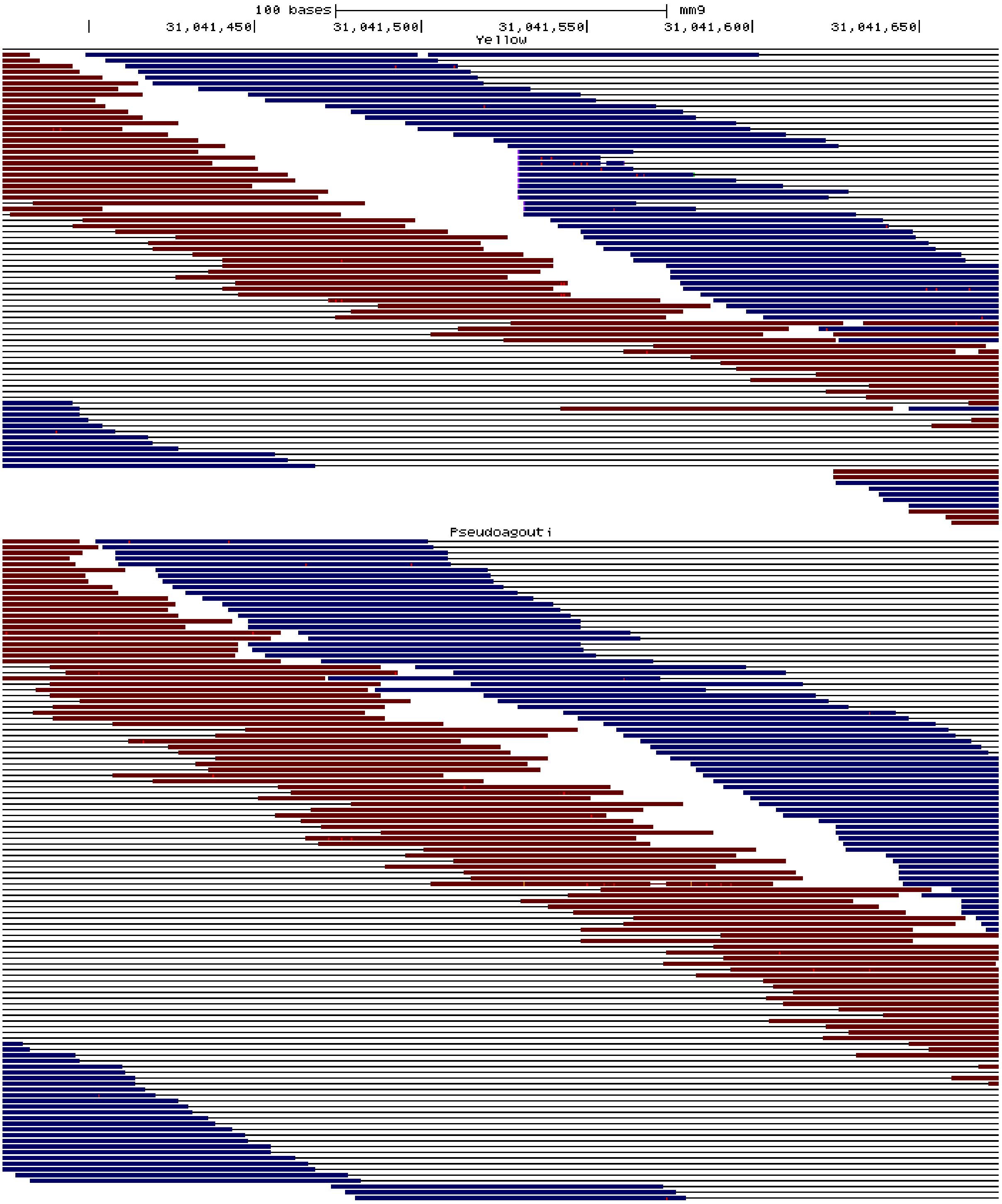

chr11:31,041,375-31,041,674

- L1 insertion
- Yellow mouse heterozygous, pseudoagouti mouse wildtype

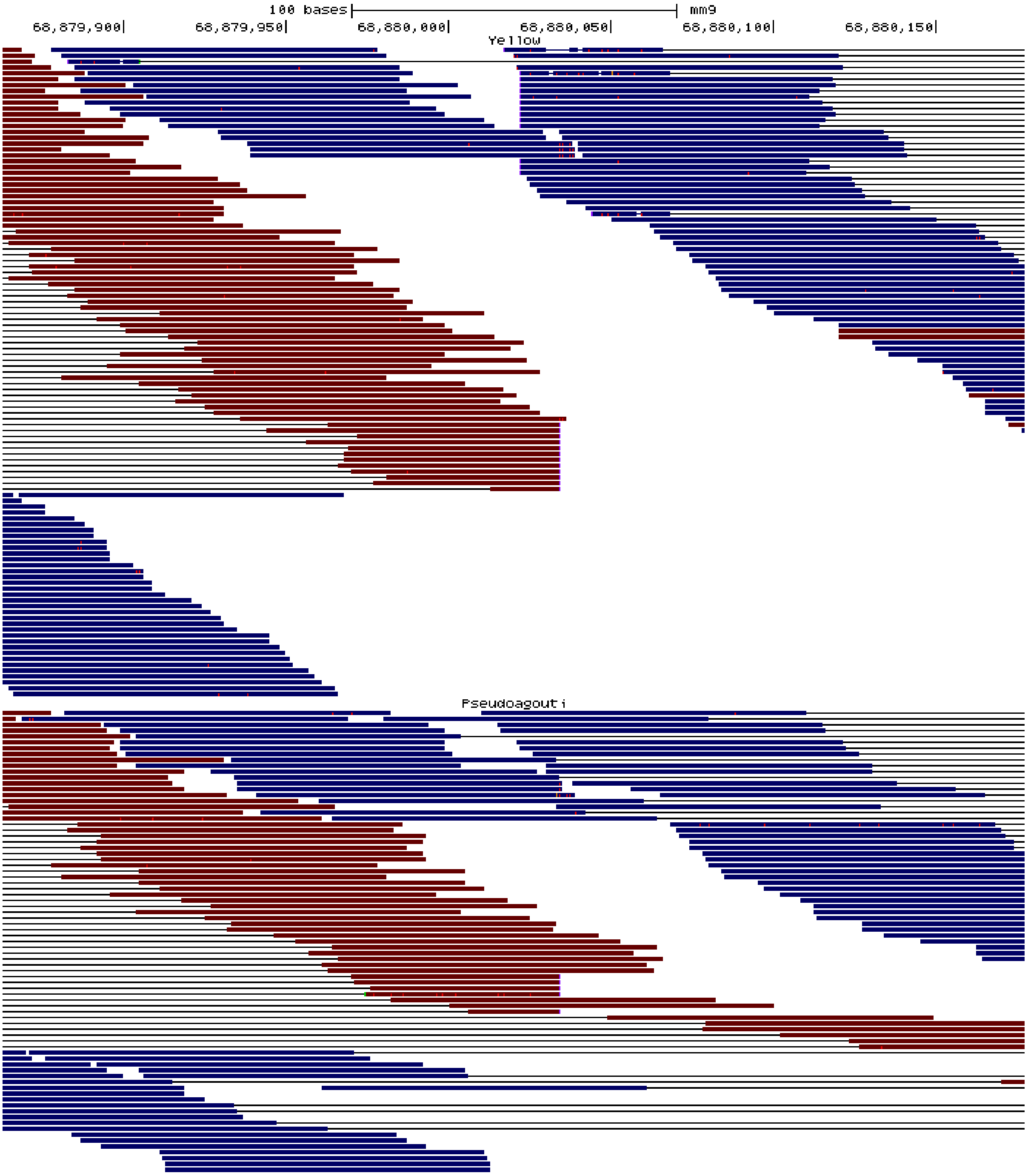

chr16:68,879,864-68,880,177

- L1 insertion
- Yellow mouse homozygous, pseudoagouti mouse heterozygous
