# Supplementary material for: Genetic and epigenetic variation among inbred mouse littermates: identification of inter-individual differentially methylated regions
Source: Epigenetics Chromatin. 2015 Dec 12;8:54. doi: 10.1186/s13072-015-0047-z (PMC4676890; doi:10.1186/s13072-015-0047-z)

a

iiDMR 61 Chr12:74938676-74939466

	Yellow	Pseudo-agouti	C57.1	C57.2	C57.3
WGBS liver DNA methylation level	0.301	0.363	0.749	0.414	0.476

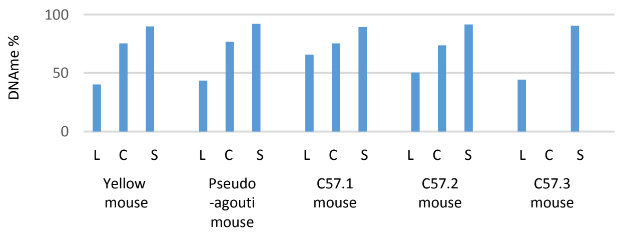

b

iiDMR 218 Chr14:65945143-65945307

	Yellow	Pseudo-agouti	C57.1	C57.2	C57.3
WGBS liver DNA methylation level	0.575	0.494	0.455	0.414	0.691

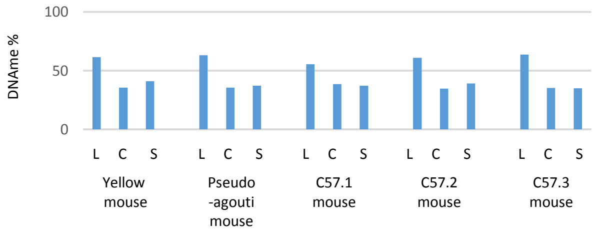

c

iiDMR 208 Chr2:103296517-103296617

	Yellow	Pseudo-agouti	C57.1	C57.2	C57.3
WGBS liver DNA methylation level	0.547	0.687	0.843	0.564	0.685

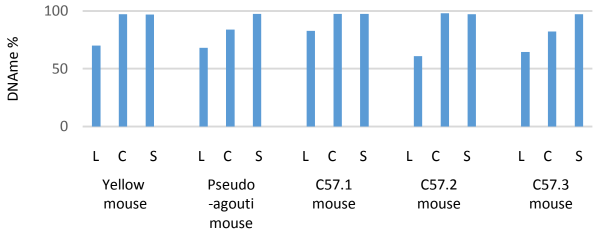

d

iiDMR 200 Chr3:109675078-109675127

	Yellow	Pseudo-agouti	C57.1	C57.2	C57.3
WGBS liver DNA methylation level	0.5	0.529	0.649	0.495	0.366

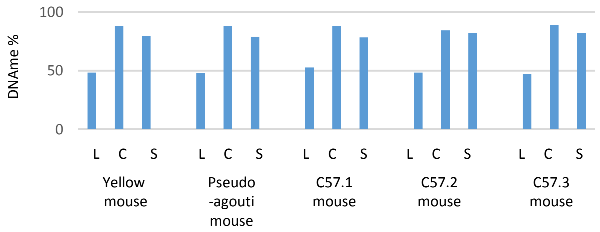

e

iiDMR 49 Chr3:122184640-122184732

	Yellow	Pseudo-agouti	C57.1	C57.2	C57.3
WGBS liver DNA methylation level	0.32	0.304	0.383	0.482	0.085

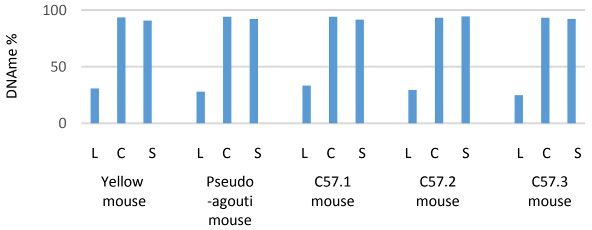

f

iiDMR 38 Chr7:20538079-20538160

	Yellow	Pseudo-agouti	C57.1	C57.2	C57.3
WGBS liver DNA methylation level	0.129	0.038	0.257	0.447	0.054

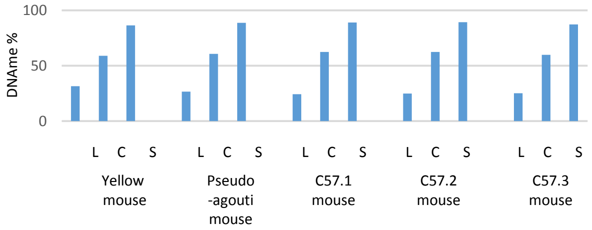

g

iiDMR 10 Chr7:53995436-53995840

	Yellow	Pseudo-agouti	C57.1	C57.2	C57.3
WGBS liver DNA methylation level	0.843	0.414	0.824	0.91	0.507

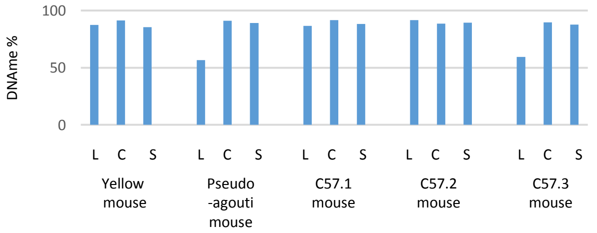

h

iiDMR 230 Chr8:124167463-124167786

	Yellow	Pseudo-agouti	C57.1	C57.2	C57.3
WGBS liver DNA methylation level	0.092	0.061	0.33	0.1	0.116

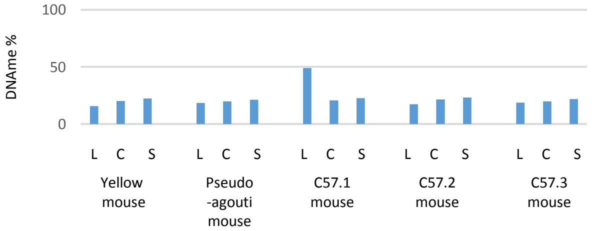

i

iiDMR 191 chr16:87512879-87512967

	Yellow	Pseudo-agouti	C57.1	C57.2	C57.3
WGBS liver DNA methylation level	0.744	0.596	0.849	0.882	0.691

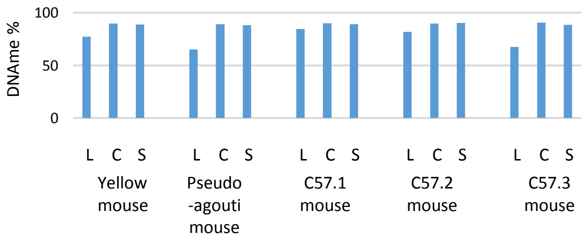

Supplement: Supplementary file 6 — 10.1186/s13072-015-0047-z Validation of DNA methylation at random non-ERV iiDMRs. WGBS weighted averages for DNA methylation values are shown for the nine loci chosen for pyrosequencing (a-i). The average pyrosequencing methylation level, from at least 4 individual CpGs from each iiDMR, is shown for liver (L), cerebellum (C) and spleen (S). DNA from these tissues was made using the five mice originally used for WGBS. Methylation levels validated in liver DNA for five of nine loci (a-e). [file 13072_2015_47_MOESM6_ESM.pdf]
